# Raw potato starch alters the microbiome, colon and cecal gene expression, and resistance to *Citrobacter rodentium* infection in mice fed a Western diet

**DOI:** 10.3389/fnut.2022.1057318

**Published:** 2023-01-10

**Authors:** Allen D. Smith, Celine Chen, Lumei Cheung, Harry D. Dawson

**Affiliations:** Diet, Genomics, and Immunology Laboratory, United States Department of Agriculture, Beltsville Human Nutrition Research Center, Agricultural Research Service, Beltsville, MD, United States

**Keywords:** resistant potato starch, microbiota, cecum, colon, gene expression, *Citrobacter rodentium*

## Abstract

Resistant starches (RS) are fermented in the cecum and colon to produce short-chain fatty acids and other microbial metabolites that can alter host physiology and the composition of the microbiome. We previously showed that mice fed a Total Western Diet (TWD) based on NHANES data that mimics the composition of a typical American diet, containing resistant potato starch (RPS), produced concentration dependent changes to the cecal short-chain fatty acids, the microbiome composition as well as gene expression changes in the cecum and colon that were most prevalent in mice fed the 10% RPS diet. We were then interested in whether feeding TWD/RPS would alter the resistance to bacterial-induced colitis caused by *Citrobacter rodentium* (*Cr*), a mouse pathogen that shares 66.7% of encoded genes with *Enteropathogenic Escherichia coli*. Mice were fed the TWD for 6 weeks followed by a 3-weeks on the RPS diets before infecting with *Cr*. Fecal *Cr* excretion was monitored over time and fecal samples were collected for 16S sequencing. Mice were euthanized on day 12 post-infection and cecal contents collected for 16S sequencing. Cecum and colon tissues were obtained for gene expression analysis, histology and to determine the level of mucosa-associated *Cr*. Feeding RPS increased the percentage of mice productively infected by *Cr* and fecal *Cr* excretion on day 4 post-infection. Mice fed the TWD/10% RPS diet also had greater colonization of colonic tissue at day 12 post-infection and colonic pathology. Both diet and infection altered the fecal and cecal microbiome composition with increased levels of RPS resulting in decreased α-diversity that was partially reversed by *Cr* infection. RNASeq analysis identified several mechanistic pathways that could be associated with the increased colonization of *Cr*-infected mice fed 10% RPS. In the distal colon we found a decrease in enrichment for genes associated with T cells, B cells, genes associated with the synthesis of DHA-derived SPMs and VA metabolism/retinoic acid signaling. We also found an increase in the expression of the potentially immunosuppressive gene, Ido1. These results suggest that high-level consumption of RPS in the context of a typical American diet, may alter susceptibility to gastrointestinal bacterial infections.

## Introduction

Resistant starches are not digested in the stomach or small intestine but are fermented in the cecum and large intestine resulting in production of microbial metabolites including short-chain fatty acids (SCFAs) and indole-3-propionate (1, 2). It is recommended that people consume approximately 15–20 g/d of RS but typical consumption from a Western diet is on average 4.9 g/day (3). Four major types of RS (RS1-4) have been defined based upon their physical and chemical properties (2) with type 2 RS (RS2) characterized by its compact granular structure that limits the accessibility of digestive enzymes (2). Consumption of RS has been shown to alter the microbiome in rodents (4–7), pigs (8–11) and humans (12–15) and is associated with changes in short-chain fatty acid levels in the cecum and colon in rodents (4, 6, 7, 16, 17), pigs (18–20) and humans (15, 21).

Although multiple studies have looked at the effect of RS on the microbiome of mice fed a high fat diet (HFD), these studies have used diets containing 45% of the calories from lard or milk fat and 8-19% sucrose by weight that do not resemble a typical Western diet. We recently demonstrated that feeding mice a rodent Total Western Diet (TWD) formulated using the 50th percentile daily intake levels for macro and micronutrients from the National Health and Nutrition Examination Survey (NHANES) (22) supplemented with different levels of resistant potato starch (RPS) led to dose dependent changes in SCFA levels of cecal contents, tissue morphology, the cecal microbiome as well as gene expression in the cecum, proximal colon (PC) and distal colon (DC) (7). The gene expression profiles identified in these studies were predicative of an increased immune response to a range of pathogens, including viruses, bacteria, and parasites.

Ulcerative colitis is an inflammatory disease of the gastrointestinal tract of unknown etiology that can cause significant morbidity and is known to be influenced by the microbiome. Colitis can also be caused by bacterial infections including *Enteropathogenic* (EPEC) and *Enterohemorrhagic Escherichia coli* (EHEC). *Citrobacter rodentium* (*Cr*) is an *Escherichia coli*-like bacterium that naturally infects mice and shares 67% of its genes with EPEC and EHEC, including genes associated with pathogenicity and virulence (23) causing disease analogous to enteropathogenic bacterial infections in humans, and thus, has served as a useful model to study infectious colitis (24). Infection of mice with *Cr* induces changes to the colon that include crypt hyperplasia, epithelial cell proliferation, an uneven apical enterocyte surface, crypt dilation, increased cellularity, and mucosal thickening (24, 25). After oral infection, *Cr* initially colonizes the cecal patch and then the colon by day 3 post-infection with peak DC bacterial load by day 7 and is typically cleared by day 21 (26). Infection with *Cr* induces a robust Th1/Th17 immune response (27).

Several studies have looked at the effect of a high-fat diet on *Cr* infections. An et al. (28) demonstrated that a lard-based Western-style diet altered the microbiome and impeded colonization and clearance of *Cr* (28). Added dietary ground flaxseed reversed the protective effect of a low-fat diet on a *Cr* infection but did not have the same effect on mice fed a high fat diet (29). The lipid content of a high-fat diet rather than total calories impacted *Cr* pathogen load and colonic pathology (30). No dietary studies with *Cr*, however, have been done in the context of a Western-style diet based on NHANES data.

*Cr* resistant and susceptible mice were initially identified in different mouse strains (31) but susceptibility in these different strains could be reversed by fecal transplants between the strains, suggesting that the microbiome was a significant factor (32, 33). More recent work has shown that even within the same strain of mice susceptibility can vary and is microbiome dependent (34). Resistance was associated with a microbiome that produced increased levels of the short-chain fatty acid (SCFA) butyrate. SCFA production is increased by diets rich in fermentable substrates including fiber and resistant starches (35). Dietary fiber was shown to be critical to preventing severe disease as fiber deficient mice had increased pathology and lethality (36, 37). Jiminez et al. (38), fed mice an AIN-93G diets containing either wheat bran or a type 2 resistant corn starch and reported that both diet alone or in combination with a *Cr* infection significantly altered the microbiome and reduced colitis severity due to *Cr* infection. An additional study by the same group showed that butyrate enemas altered the microbiome and reduced *Cr-*induced colitis (39). These studies suggest that fermentable substrates that produce bacterial metabolites such as SCFAs can influence the outcome of *Cr* infections. To investigate this phenomenon further we conducted studies examining the effect of feeding a Western-style diet based on NHANES data containing different levels of RPS on subsequent *Cr* infections. We found that mice fed the10% RPS had increased colonization and colon pathology compared to TWD fed mice and that both infection and diet had major impacts on the microbiome and gene expression in the cecum and DC.

## Materials and methods

### Animals and diet

C57BL/6 mice were originally purchased from Charles River (Frederick, MD) and bred in house. Mice were housed in ventilated filter-top cages at the USDA BHNRC animal facility under 12-h light/dark cycle. Timed breedings were set up and offspring were weaned at 3–4 weeks of age. Breeding pairs were fed rodent chow (Teklad 2020X, Frederick, MD). Only female offspring were used in these experiments. After weaning, female mice were group housed (4–5/cage) placed on the TWD [Supplementary Table 1, Envigo, Madison, WI] (22). After feeding mice the TWD for 6 weeks, mice were divided into one of 4 diets dietary treatment groups depending on the experiment; TWD or TWD in which some of the corn starch was replaced with RPS (Ingredion, Westchester, IL) at 2, 5, or 10% w/w RPS for an additional 3 weeks as described previously (7). Mice were periodically weighed. All experiments were approved by the USDA-ARS Beltsville Institutional Care and Use Committee.

### *Citrobacter rodentium* infections

After the dietary regimens described above were completed, mice were infected with *Cr* and maintained on their respective diets until the end of the experiment. The *Cr* strain used was a nalidixic acid-resistant mutant of strain DBS100 (ATCC 51459). A culture of *Cr* was incubated overnight at 37°C with shaking. The following morning the culture was expanded and grown to an OD_600_ of approximately 1.5, harvested by centrifugation and resuspended in LB broth. Mice were infected with 2.5–5.0 X 10^9^ cfu by oral gavage after a 4–6 hour fast and the dose confirmed by retrospective plating. Uninfected controls received LB broth.

### Sample collection and processing

After infection, mice were periodically weighed, and fecal pellets were collected to measure fecal shedding of *Cr* or for 16S analysis. For determining fecal *Cr* shedding, fecal pellets were homogenized in LB broth and serial dilutions plated on LB agar plates with 50 μg/mL nalidixic acid. Results were expressed as cfu/g colon feces. Mice that were not productively infected as measured by low or no fecal load early in infection (days 4–6) were removed from all analyses to ensure uniform infection kinetics. Mice were euthanized on day 12 post-infection to obtain tissues or cecal contents for analyses by i.m. injection of 320 mg/kg ketamine/1,000 mg/kg xylazine mix followed by exsanguination. The colon was excised, and the length measured. The terminal six cm of the colon was then taken, the colonic contents removed, the tissue weighed, and subdivided into one-centimeter portions that were fixed in 4% formalin for histology or snap frozen for gene expression analysis. The remaining section was homogenized in PBS and serial dilutions plated on LB agar plates with 50 μg/mL nalidixic acid to determine the *Cr* load in the colon tissue. Results are expressed as cfu/g colon tissue. Day 11 fecal or D12 colon samples with a colony count of 0 for duplicate plates, a colony count value of 0.5 was assigned that represents the limit of detection for statistical and graphical purposes. For measuring fecal pH, fecal pellets were weighed and homogenized in 5 volumes of water, centrifuged to removed debris and the pH of the supernatant measured.

### Histology

Equivalent one cm sections from the cecum or DC were obtained on day 12 post-infection. The tissues were fixed in buffered 4% paraformaldehyde. The sections were then paraffin embedded and 5 μm sections were cut and stained with hematoxylin and eosin (H&E). The slides were coded and sections were evaluated for damage to the surface epithelium (0–4), degree of hemorrhaging (0–4), loss of crypt architecture (0–4), and the presence of an inflammatory cell infiltrate (0–4). Crypt depth was measured using a Nikon Eclipse E800 microscope and Nikon NIS-Elements software V4.6. The crypt depth for each mouse was determined by averaging multiple measurements of well-oriented crypts. These average values were then used for statistical analysis to determine differences between dietary groups.

### 16S sequencing of cecal contents

DNA was isolated from fecal pellets or cecal contents using the *Quick*-DNA Fecal/Soil Microbe Microprep Kit (Zymo, Irvine, CA) following the manufacturer's instructions and then further purified using the DNA Clean and Concentration kit (Zymo, Irvine, CA). The DNA concentration of the samples was quantified using a Quant-it PicoGreen dsDNA kit (Invitrogen, Waltham, MA). Bacterial DNA samples were submitted to the Michigan State University RTSF Genomics Core for targeted amplicon library preparation and sequencing. The V3-V4 hypervariable regions of the 16S rRNA gene was amplified using indexed, Illumina compatible primers 341f/806r as described (40) with the exception that the V3 flanking primer 341f was substituted for the V4 primer 515f. The pooled libraries were loaded into an Illumina MiSeq v2 500 cycle reagent cartridge. The FASTQ files with raw data were submitted to the National Center for Biotechnology Information (NCBI) Sequence Read Archive (SRA) under the BioProject ID: PRJNA757013.

The 16S rRNA tag data curation and processing were performed using the CLC Microbial Genomics Module (QIAGEN Bioinformatics, Redwood City CA) following its standard OTU clustering workflow as previously described (7). The processed contigs were subsequently aligned to the SILVA SSU database from release v138.1 (41) clustered into Operational Taxonomic Units (OTUs) at 97 percentage similarity. Alpha and beta diversity were measured using the MUSCLE tool (42) to reconstruct the phylogenetic tree by a Maximum Likelihood approach. PERMANOVA (43) was performed to measure the effect size and significance of beta diversity. To examine the changes induced by the RPS treatments, PCA and hierarchical clustering were performed with the OTU or taxon-specific abundance profiles. Data distribution induced by the RPS treatments was visualized by PCA analysis in JMP Genomics 10 with default settings. Linear discriminant analysis effect size [LEfSe (44)] analysis was performed to identify RPS-specific biomarkers.

### RNASeq analysis of cecum and distal colon tissue

RNA from the cecum and DC was isolated using Tri-Reagent (Zymo, Irvine, CA) and Purelink RNA kits (Invitrogen, Carlsbad, CA). The samples were further purified using RNA Clean and Concentrate columns (Zymo, Irvine, CA) and the samples were submitted to the Michigan State University RTSF Genomics Core facility for sequencing. Libraries were prepared using Illumina Stranded mRNA Prep, Ligation kit with IDT for Illumina Unique Dual Indexes following manufacturer's recommendations. Completed libraries were quantified using a combination of Qubit dsDNA HS and Agilent 4200 TapeStation HS DNA1000 assays. Libraries were pooled in equimolar amounts for multiplexed sequencing, and the pool quantified using the Invitrogen Collibri Quantification qPCR kit.

The pools were loaded onto an Illumina NovaSeq S2 flow cell and sequencing was performed in 1x100bp single read format using a NovaSeq 6000 v1.5 100 cycle reagent kit. Base calling was done by Illumina Real Time Analysis (RTA) v3.4.4 and output of RTA was demultiplexed and converted to FastQ format with Illumina Bcl2fastq v2.20.0. The FASTQ files with raw data and the gene expression profiles were submitted to the National Center for Biotechnology Information (NCBI) Sequence Read Archive (SRA) under the BioProject ID: PRJNA757013.

Sequences were processed to determine gene expression levels. Before sequence alignments performed by the CLC Genomics Workbench version 20.01 (QIAGEN Bioinformatics, Redwood City CA), nucleotides below Q30 or reads containing more than two ambiguous nucleotides were removed. To calculate gene expression in counts, reads were mapped to the Mus musculus genome assembly GRCm39. Transcriptomes were built from the alignments.

Differentially expressed genes (DEGs) were determined to be genes that were up or down regulated > 1.5-fold at a false discovery rate (FDR) adjusted *p* < 0.05. We functionally annotated DEGs using our Porcine Translational Research Database (45). The database serves to translate data found in rodents or pigs to human. VENN analysis of DEGs within individual groups was conducted using the online tool, VENNY 2.1 (https://bioinfogp.cnb.csic.es/tools/venny/index.html). Pathway analysis on DEGs was conducted using the online tool, DAVID (https://david.ncifcrf.gov) (46) using Knowledgebase v2022q2. Data was queried against the embedded Reactome (47) and KEGG database. VENN analysis was also conducted on differentially expressed (at a FDR adjusted *p* < 0.05%) Reactome and KEGG pathways identified by DAVID.

### Statistical analyses

Data was analyzed using a student's *t*-test, one-way or two-way ANOVA were used were applicable. Data was transformed as necessary to achieve equal variance and normality. In cases where equal variance and normality could not be met, a Welch's *t*-test, Mann-Whitney Rank Sum test or a Kruskal-Wallis One Way Analysis of Variance on Ranks were run. Histopathology scores were analyzed by a Mann-Whitney Rank Sum test. For determining treatment effects in microbiota data obtained from 16S sequencing, a two-way ANOVA was carried out using JMP Genomics 10. Pairwise comparisons between treatments were conducted with the Student's t-test. Transcriptomes were subjected to differential expression analysis with DESeq2.

## Results

### Effect of RPS and infection on body weights, fecal pH, and tissue/body weight ratios

**Figure 1:**
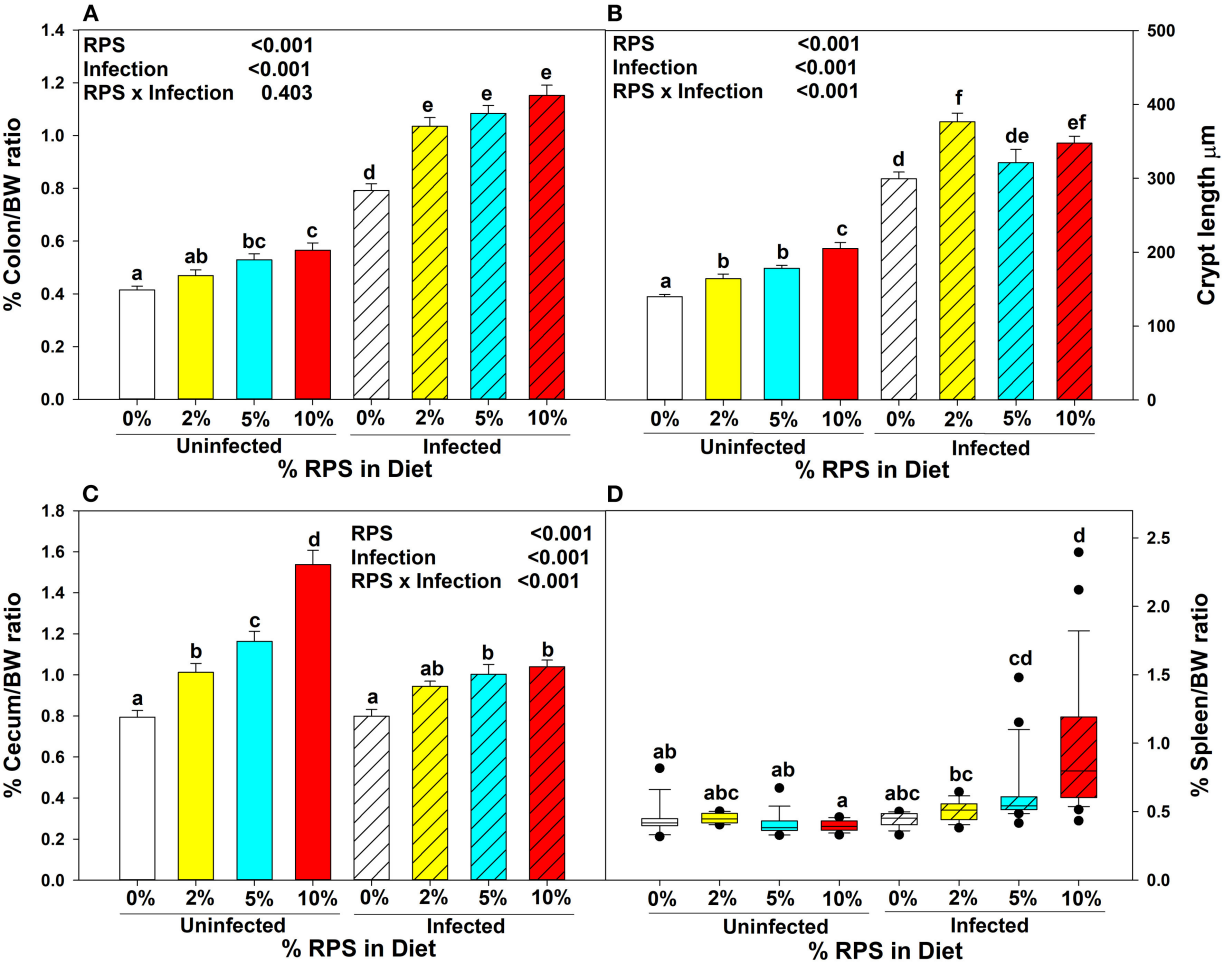
Dietary RPS and *Cr* infection alter tissue/body weight ratios and crypt length. Mice were fed the TWD for 6 weeks and then switched to RPS containing diets for an additional 3 weeks. A subset of mice were infected with *Cr* and euthanized 12 days post-infection. The effect of feeding RPS and *Cr* infection on %Tissue/bodyweight ratios were calculated for **(A)** colon, **(C)** cecum and **(D)** spleen. Data is from four independent experiments combined, bars are mean ± SEM, *n* = 14-26/group. **(B)** The crypt length of uninfected and infected mice fed the different levels of RPS were measured on well-oriented crypts. Data from two independent experiments was combined, *n* = 5–10, bars are mean ±SEM. Data in **(A–C)** were analyzed by two-way ANOVA followed by multiple comparisons using the Holm-Sidak method. Data in **(D)** were analyzed by a one-way ANOVA on ranks followed by multiple comparisons using Dunn's Method. In all panels, bars with different letters are significantly different *p* < 0.05.

Three to 4 week-old mice were placed on the TWD for 6 weeks to allow sufficient time for the effects of feeding the TWD on metabolism, growth rate and microbiome changes to stabilize (48). Subsets of mice were left on the basal TWD or switched to 2, 5, or 10% RPS-containing diets for an additional 3 weeks and then infected with *Cr*. Mice remained on their respective diets until euthanasia at 12 days post-infection. Body weights were not affected by feeding different levels of resistant starch diets for three weeks (Supplementary Figure 1A). In general, infection had little to no effect on weight gain (Supplementary Figure 1B). We and others have shown that feeding RPS to uninfected mice can result in an increase in colon and cecum weight (49, 50) and that was confirmed in this study (Figures 1A, C). The increase in colon weight correlated with a RPS dose-dependent increase in DC crypt length (Figure 1B).

*Cr* infection induces colonic hyperplasia (51). We also found that %colon/BW ratios increased substantially in infected RPS fed mice but the differences between infected mice fed different levels of RPS were not as pronounced as seen in uninfected mice (Figure 1A) and this was reflected in the crypt length as well (Figure 1B). Infection with *Cr* reduced the dose dependent increase in cecum weight (Figure 1C). We previously showed that feeding mice our TWD/RPS diets decreased fecal pH in a dose dependent manner (7). To see if infection altered the decrease in fecal pH, the pH of fecal samples, (D11 post-infection) collected from uninfected and infected mice fed the basal TWD or the 10% RPS diet, was determined. As seen before, feeding mice a 10% RPS diet resulted in a decrease in fecal pH, but this was unaltered by *Cr* infection (Supplementary Figure 2). Spleen weight was not affected by feeding RPS in uninfected mice or in infected mice fed 0 or 2% RPS diet but was increased to some extent in mice fed the 5% RPS diet and was substantially increased in mice fed the 10% RPS diet (Figure 1D) suggesting systemic involvement in infected mice fed higher levels of RPS.

### Effect of RPS on the Cr colonization and histopathology

**Figure 2:**
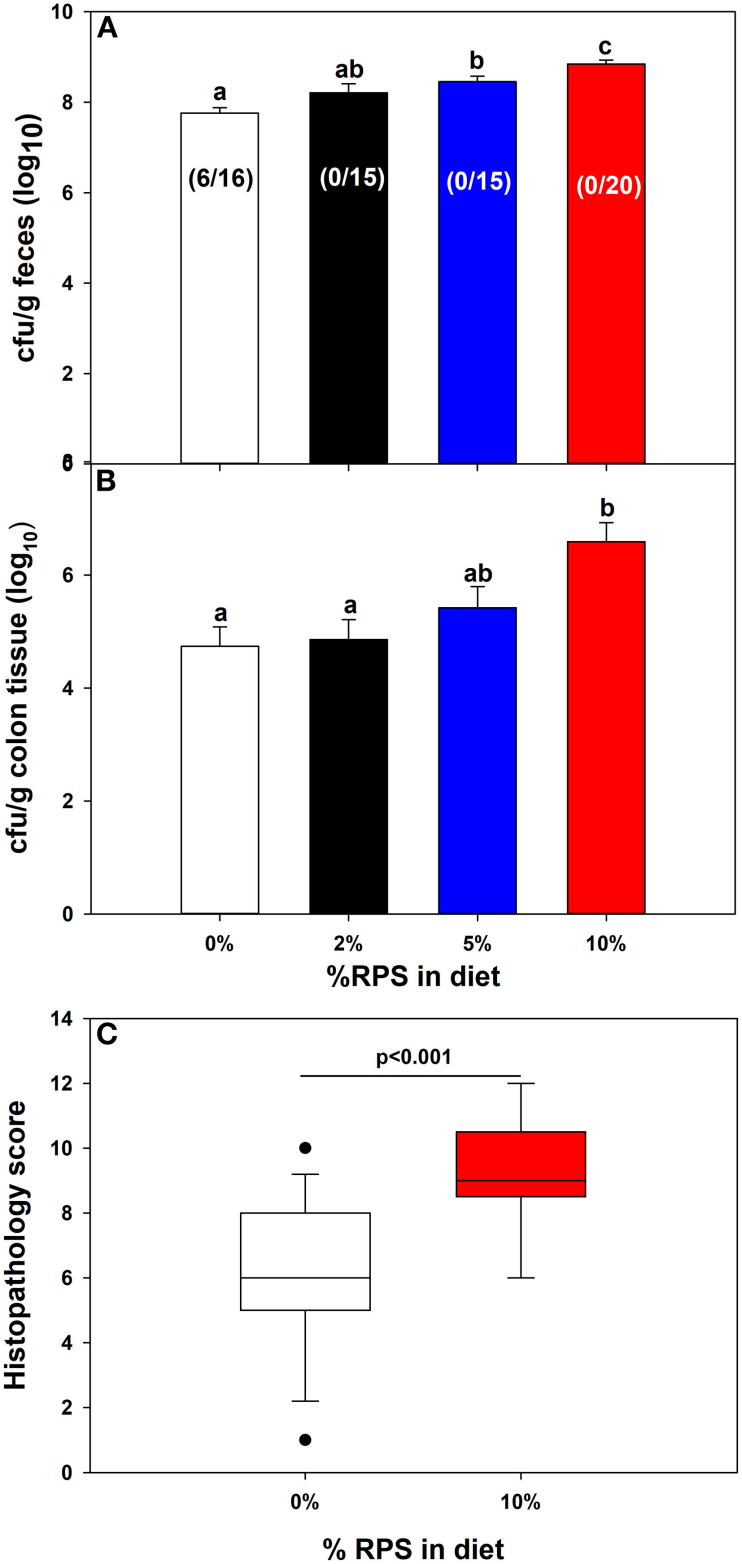
Feeding RPS altered colonic colonization of *Cr* and histopathology. Mice were fed the TWD for 6 weeks followed by the RPS diets for 3 weeks and then infected with *Cr*. **(A)** Fecal samples were collected at 4 days post-infection and the *Cr* fecal burden determined. Results for productively infected mice are expressed as the mean ± SEM of log_10_ transformed *Cr* colony forming (CFU)/g feces, Data from three independent experiments combined (*n* = 16-20). Shown in parentheses are the # of unproductively infected mice over the total mice inoculated. **(B)** The amount of mucosa adherent *Cr* was determined on colon sections obtained 12 days post-infection. Results are the mean ± SEM of log_10_ transformed *Cr* colony forming (CFU)/g colon tissue. Data is from four independent experiments combined using productively infected mice only *n* = 14–26/group. **(C)** Histopathology scores for infected mice fed the 0% RPS diet vs. the 10% RPS diet. Results of the Mann-Whitney Rank Sum Test are displayed as box plots. Data from three independent experiments were combined, *n* = 13–17 mice/group. Groups with different letters are significantly different, *p* < 0.05.

Fecal excretion of *Cr* was monitored over time. Feeding increasing amounts of dietary RPS resulted in softer stools that was further aggravated by *Cr* infection. On day four post-infection, a subset of mice (38%) fed the TWD were not productively infected with low or almost no detectible colonization and the remaining TWD mice had lower levels of fecal *Cr* than mice fed the RPS containing diets which all were productively infected and had increasing levels of fecal *Cr* excretion in a dose dependent manner (Figure 2A). On day 12 post-infection, mice were euthanized, and the amount of mucosa-associated *Cr* determined. Only mice fed the 10% RPS diet had increased *Cr* colonization compared to mice fed the basal 0% RPS diet (Figure 2B) and was further supported by an increased level of fecal *Cr* excretion at day 11 post-infection in mice fed the 10% RPS diet compared to infected mice fed the basal TWD (Supplementary Figure 3). The increased colonization of the colon at day 12 post-infection in mice fed the 10% RPS diet was associated with the increase in spleen size (Figure 1C) suggesting that the mucosal barrier might be compromised in infected mice fed the 10% RPS diet. Examination of H&E-stained sections from infected mice fed the 0% and 10% RPS diet confirmed that infected mice fed the 10% RPS diet had increased colon pathology compared to infected mice fed the basal diet (Figure 2C).

### Effect of RPS and Cr infection on the cecal and fecal microbiome

**Figure 3:**
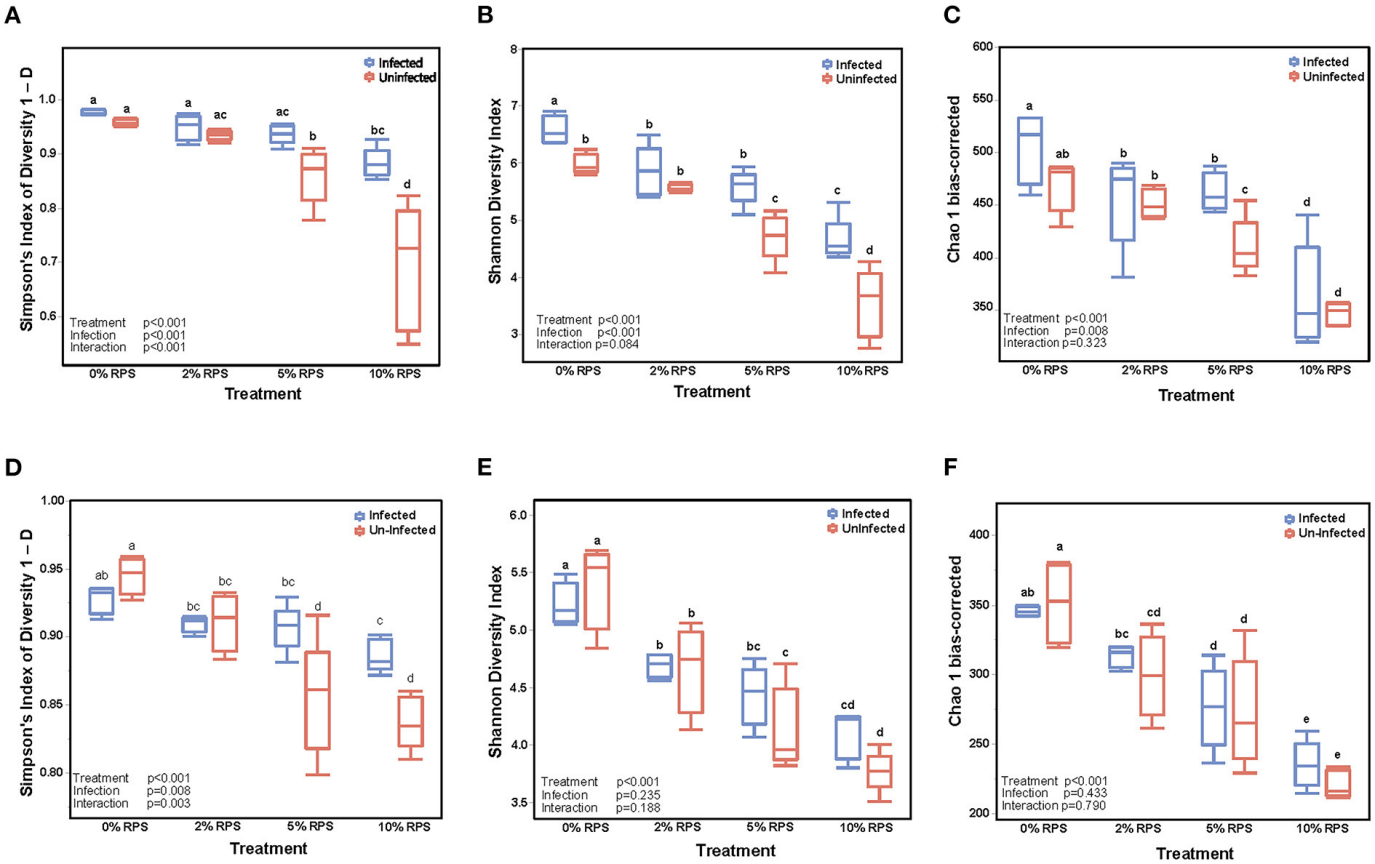
Feeding RPS decreases the α-diversity of fecal and cecal microbiomes and the relative abundance of genera and this is partially reversed by *Cr* infection. α-diversity was determined on cecal samples collected at 12 days post-infection **(A–C)** and on fecal samples collected 6 days post-infection **(D–F)**. *n* = 4–5/group. Groups with different letters are significantly different, *p* < 0.05.

The cecum is the primary site of fermentation in mice and is the initial site of colonization by *Cr* but by day 12 post-infection the DC is the primary site of colonization. Nevertheless, we investigated the effect of diet and infection on both the cecal and fecal microbiome. The α-diversity of infected and uninfected cecal samples fed different dietary levels of RPS were measured by the Simpson Index (Figure 3A), the Shannon Diversity Index (Figure 3B), and the Chao-1 bias-correct (Figure 3C). All 3 indices showed a significant effect of both treatment (dietary RPS) and infection on α-diversity but only the Simpson Index showed a significant interaction between treatment and infection. As we previously reported (7), α-diversity in uninfected mice declined with increasing dietary RPS and *Cr* infection partially reversed this trend. In contrast, α-diversity in the day 6 fecal samples was primarily driven by dietary treatment (Figures 3E, F) with only the Simpson Index (Figure 3D) showing an effect of infection and an interaction between diet and infection.

**Figure 4:**
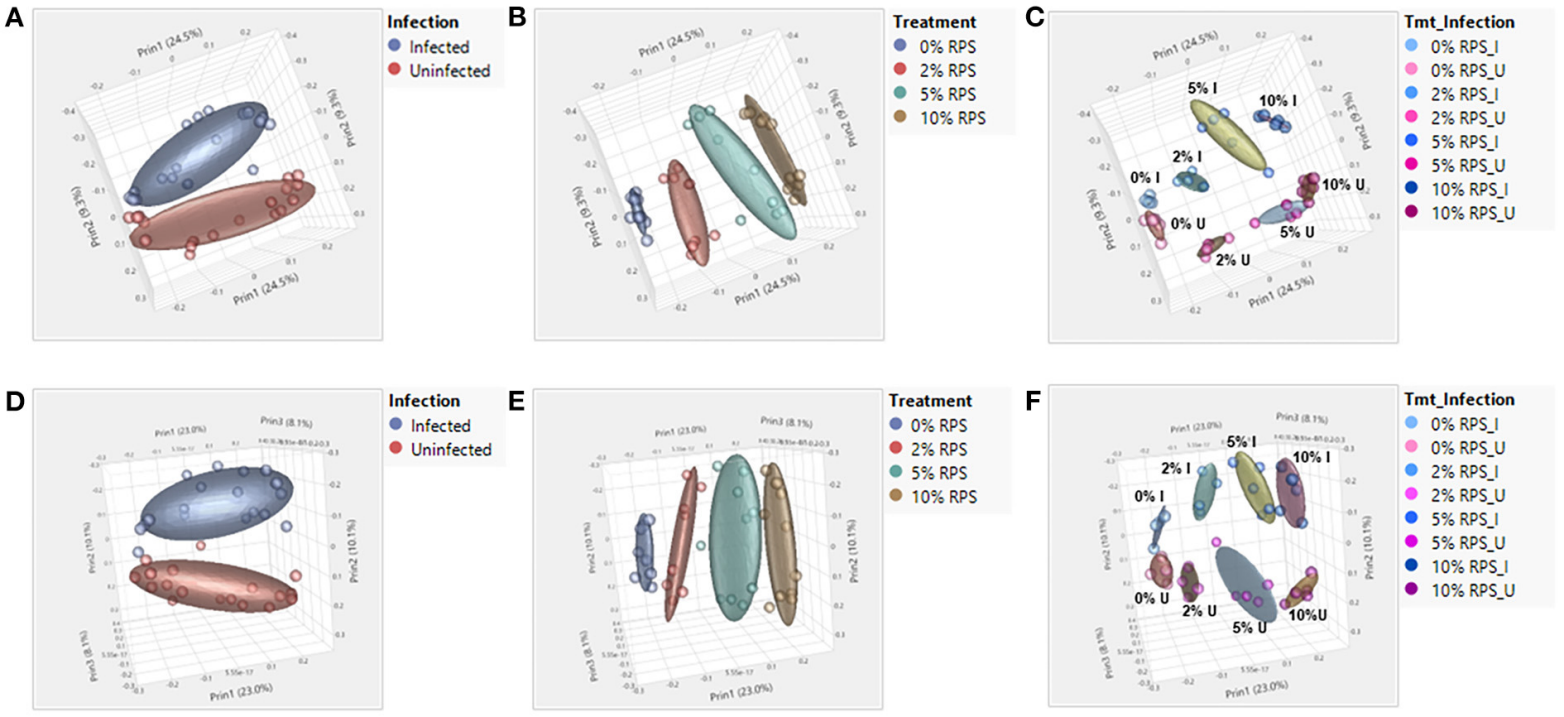
Both feeding RPS diets and *Cr* infection leads to distinct grouping by PCA analysis in fecal and cecal samples. PCA analyses were performed using JMP Genomics and analyzed based on diet, infection, and the combination of the two. *n* = 4–5 mice/group, eight groups, four uninfected and four infected. **(A–C)** are for cecum samples and D-F for fecal samples. **(A, D)** PCA illustrating the effect of infection on the microbiome regardless of dietary intake; **(B, E)** PCA illustrating the effect of dietary RPS levels only on the microbiome; **(C, F)** PCA illustrating the effect of diet and infection on the microbiome.

A breakdown of the variance components for cecal and fecal samples at the genus taxonomic level indicated that diet was the primary component with much smaller contributions from infection and diet-infection interactions for cecal samples (Supplementary Figure 4A) and contrasts with the variance components for fecal samples where infection and treatment were nearly identical and there was a much smaller contribution from diet-infection interactions (Supplementary Figure 4B). The increase in the contribution due to infection in fecal samples is indicative of the high level of *Cr* colonization of the DC at day 6 post-infection and is reflected in the greater distance between infected vs. uninfected groups in the fecal PCA plot (Figure 4D) compared to the cecal PCA plot (Figure 4A). Diet effects on the PCA plots were similar for cecal and fecal samples (Figures 4B, E). PCA plots looking at the effect of both diet and infection produced plots in which the samples segregated into the eight groups, but the day 6 fecal samples also formed subclusters based on infection status that was more pronounced than in the cecal samples (Figures 4C, F).

**Figure 5:**
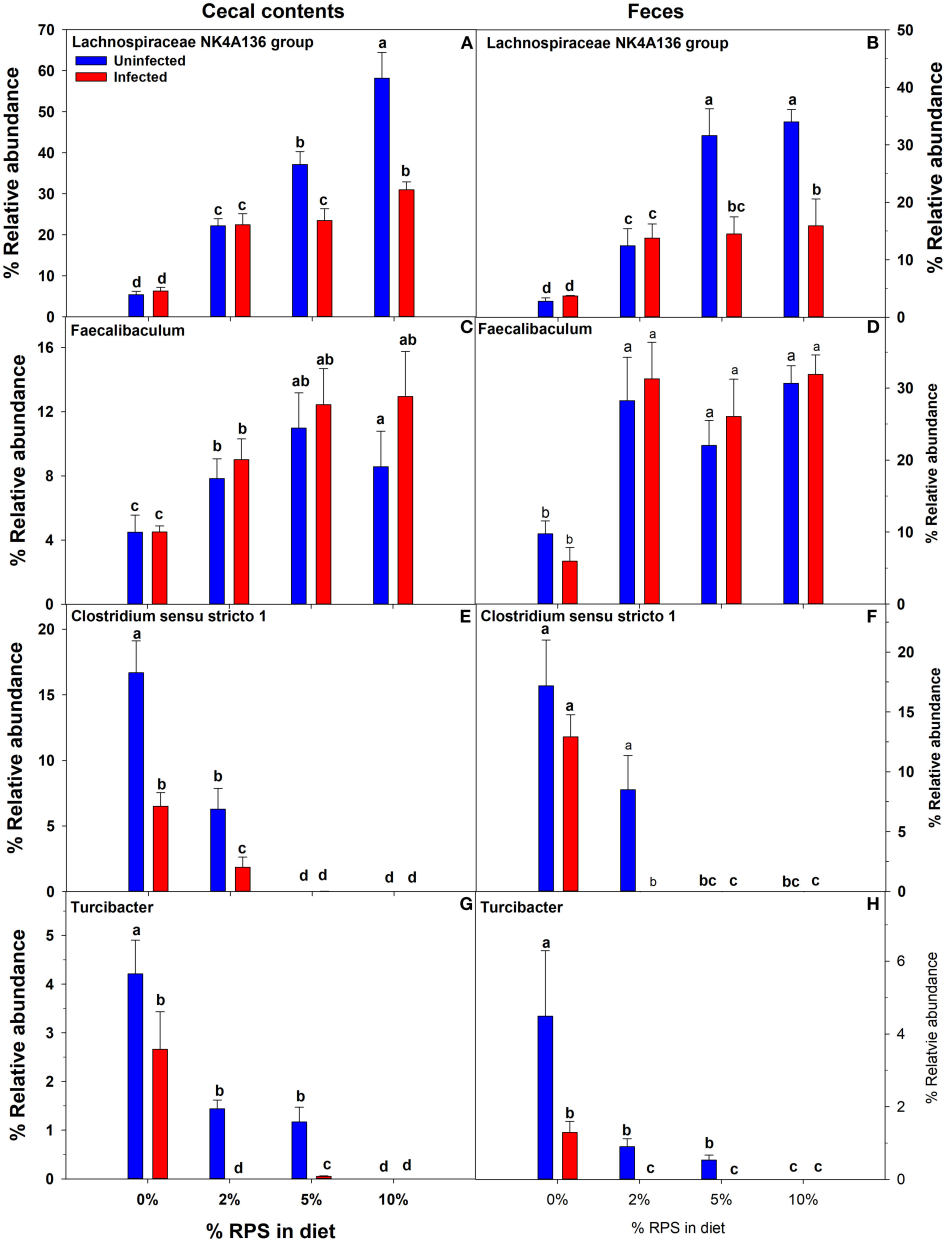
Both feeding RPS diets and *Cr* infection altered the relative abundance of various genera in cecal and fecal samples. Cecal samples obtained 12 days post-infection and fecal samples obtained 6 days post-infection from uninfected and infected mice were subjected to 16S sequencing and analysis. Shown are examples were diet and infection altered the relative abundance of **(A, B)** *Lachnospiraceae NKA136 group*, **(C, D)** *Faecalibaculum* **(E, F)** *Clostridium sensu stricto 1*, and **(G, H)** *Turcibacter*. Bars are means ± SEM. Groups with different letters are significantly different by two way ANOVA, *p* < 0.05, *n* = 4–5/group.

Confirming what we had previously shown in uninfected mice (7), a stack plot of the relative abundance of taxa at the genus level in cecal contents showed a large increase to nearly 60% relative abundance of the *Lachnospiraceae NK4A136 group* at 10% dietary RPS, becoming the dominant genera in uninfected mice (Supplementary Figure 5A). This large increase in the *Lachnospiraceae NK4A136 group* was reduced by half by *Cr* infection showing a significant treatment X infection interaction (Supplementary Figure 5A; Figure 5A; Table 1). A D6 post-infection stack plot showed that in uninfected fecal samples the *Lachnospiraceae NK4A136 group* relative abundance also increased in response to dietary RPS but peaked at ~35% compared to nearly 60% in the cecal contents and was also decreased by about half by *Cr* infection, again showing a significant treatment X infection interaction (Supplementary Figure 5B; Figure 5B; Tables 1, 2). Another genera, the *Faecalibaculum*, relative abundance also increased significantly in response to dietary RPS in uninfected cecal and fecal samples (Figures 5C, D; Tables 1, 2) but was substantially higher in feces from mice fed the 10% RPS diet, about 30% compared to about 9% in the cecal contents. The increase in *Faecalibaculum* in uninfected fecal samples was accompanied by a corresponding decrease in the maximum level of fecal *Lachnospiraceae NK4A136*. Thus, in the uninfected fecal samples, the *Lachnospiraceae NK4A136 group* and *Faecalibaculum* account for about 65% of the relative abundance in uninfected fecal samples (Figure 5; Supplementary Figure 5B; Table 2). *Faecalibaculum* relative abundance was not significantly affected by *Cr* infection in either fecal or cecal samples. Two other genera that showed significant treatment, infection, and treatment X infection interactions in both the cecum and feces were *Clostridium sensu stricto 1* and *Turcibacter*, both of which declined in response to increasing levels of RPS but were altered by infection as well (Figures 5E–H; Tables 1, 2). In general, the diet induced trends amongst genera of uninfected groups were similar between D6 fecal and D12 cecal samples although the relative abundance values may differ (Tables 1, 2). One exception was *Odoribacter* whose relative abundance levels increased in response to increasing dietary RPS in uninfected cecal but not in fecal samples and was reduced by infection in fecal but not cecal samples. Another exception was *Erwinia*, a Gram-negative bacteria related to *E. coli, Shigella, Salmonella*, and *Yersinia* (52), which was not present in uninfected cecal or fecal samples or in infected cecal samples but was present in fecal samples from infected mice and increased in response to increasing dietary RPS (Tables 1, 2).

**Table 1:** Effect of diet and *C. rodentium* infection on various genera with relative abundance above 0.1% in cecal contents from uninfected and infected mice obtained at day 12 days post-infection^1^.

**Treatment**	**Uninfected**				**Infected**				**Pooled SEM**			Treatment *infection
**Sample ID**	**0%**	**2%**	**5%**	**10%**	**0%**	**2%**	**5%**	**10%**		**Treatment**	**Infection**	
[Eubacterium] coprostanoligenes group	0.11^a^	0.03^b^	0.00^d^	0.00^d^	0.03^b^	0.01^c^	0.00^d^	0.00^d^	0.05	2.47E–14	8.43E–04	8.05E–03
Akkermansia	6.47^b^	11.29^a^	9.78^a^	6.92^a^	7.17^b^	7.91^b^	9.55^ab^	10.89^a^	3.44	1.33E–03	3.28E–02	2.11E–01
Alistipes	12.3^a^	8.92^abc^	7.77^ab^	2.47^d^	9.09^bcd^	7.25^cd^	4.61^d^	3.76^d^	3.37	3.29E–03	2.37E–04	1.26E–01
Anaerotruncus	0.04^bc^	0.02^c^	0.02^bc^	0.01^bc^	0.08^bc^	0.11^b^	0.25^a^	0.26^a^	0.13	6.27E–02	1.87E–06	6.08E–02
Azospirillum sp. 47_25	0.07e	0.16^cd^	0.3^ab^	0.20^abc^	0.14de	0.22^bcd^	0.20^cd^	0.81^a^	0.38	2.37E–04	8.93E–01	1.06E–01
Bacteroides	0.49^d^	0.55^cd^	1.73^b^	2.73^a^	0.57^d^	0.87^d^	1.39^bc^	6.82^a^	1.20	4.70E–12	7.36E–01	1.75E–01
Bifidobacterium	0.32^b^	1.98^a^	1.00^a^	2.30^a^	0.23^b^	1.59^a^	3.95^a^	3.03^a^	1.61	1.20E–07	9.80E–01	2.40E–01
Bilophila	2.68^a^	1.54^ab^	1.03^ab^	0.38^cd^	2.28^ab^	2.49^ab^	1.38^bc^	0.37^d^	0.99	2.12E–06	1.20E–01	1.87E–01
Blautia	6.13^a^	2.87^ab^	1.11^bc^	0.62^cd^	3.72^bc^	2.14^bc^	2.26^bc^	0.42^d^	1.87	2.18E–04	2.16E–02	1.74E–01
Catenibacterium	0.20^abc^	0.29^ab^	0.10^c^	0.22^a^	0.19^abc^	0.10^bc^	0.22^abc^	0.17^abc^	0.15	2.29E–01	2.30E–01	1.29E–01
Citrobacter	0.00^b^	0.00^b^	0.00^b^	0.00^b^	0.00^b^	0.00^b^	0.00b*	0.07^a^	0.04	1.96E–04	1.69E–04	1.96E–04
Clostridium sensu stricto 1	16.69^a^	6.29^b^	0.00^d^	0.00^d^	6.51^b^	1.86^c^	0.02^d^	0.01^d^	3.20	5.66E–20	9.63E–02	2.73E–02
Desulfovibrio	0.62^ab^	0.88^a^	0.73^a^	0.52^a^	0.97^ab^	0.56^bc^	0.5^ab^	0.20^c^	0.43	5.63E–01	4.60E–04	4.47E–02
Dubosiella	0.03^d^	0.07^bcd^	0.03^cd^	0.11^a^	0.04^cd^	0.05^bcd^	0.10^ab^	0.08^abc^	0.05	9.92E–04	6.36E–01	3.24E–02
Eisenbergiella	0.15^ab^	0.04^bc^	0.02^c^	0.02^c^	0.22^a^	0.04^c^	0.02^c^	0.00^d^	0.05	1.16E–06	1.08E–01	1.73E–01
Faecalibaculum	4.49^c^	7.84^b^	10.97^ab^	8.57^a^	4.50^c^	9.01^b^	12.43^ab^	12.95^ab^	5.37	3.17E–06	5.96E–01	9.54E–01
GCA-900066575	0.99^ab^	0.73^ab^	0.46^b^	0.39^ab^	1.54^a^	1.14^ab^	1.17^ab^	0.78^ab^	0.56	3.60E–01	1.20E–01	6.39E–01
Intestinimonas	0.11^ab^	0.07^ab^	0.10^a^	0.08^a^	0.14^ab^	0.13^ab^	0.06^b^	0.06^ab^	0.10	8.40E–01	7.60E–02	1.57E–01
Lachnoclostridium	2.12^b^	1.60^b^	0.60^d^	0.53^cd^	4.67^a^	2.44^b^	1.66^bc^	1.34^bc^	0.97	1.50E–05	4.64E–04	6.42E–01
Lachnospiraceae NK4A136 group	5.41^d^	22.22^c^	37.11^b^	58.16^a^	6.30^d^	22.46^c^	23.51^c^	30.97^b^	8.80	5.28E–16	1.83E–05	2.78E–03
Lachnospiraceae UCG-001	0.11^a^	0.14^a^	0.03^b^	0.02^b^	0.13^ab^	0.14^a^	0.08^ab^	0.01^c^	0.05	2.99E–07	1.41E–01	7.90E–03
Lachnospiraceae UCG-006	0.43^a^	0.15^abc^	0.14^ab^	0.10^ab^	0.53^a^	0.42^ab^	0.14^bc^	0.09^c^	0.22	6.61E–03	2.37E–01	9.90E–02
Lachnospiraceae UCG-008	0.21^ab^	0.04^d^	0.02^d^	0.02^cd^	0.47^a^	0.13^bc^	0.04^d^	0.03^d^	0.06	1.73E–07	1.17E–01	1.64E–01
Lactobacillus	3.87^a^	2.41^a^	0.53^b^	0.19^b^	7.22^a^	2.61^a^	0.66^b^	1.70^a^	2.38	6.40E–06	6.09E–02	1.97E–02
Lactococcus	0.05^b^	0.05^ab^	0.03^b^	0.02^b^	0.16^a^	0.07^ab^	0.04^b^	0.05^b^	0.05	9.68E–02	4.50E–01	1.43E–01
Mucispirillum	1.25^bc^	0.7^cd^	0.43^d^	0.30^d^	2.49^b^	1.28^bc^	1.88^b^	3.48^a^	1.02	1.38E–01	1.75E–07	3.05E–04
Odoribacter	0.92^bcd^	3.73^a^	4.30^a^	2.84^abc^	1.23^d^	3.44^ab^	3.09^abc^	2.05^cd^	2.49	5.16E–03	7.04E–02	6.11E–01
Oscillibacter	1.20^ab^	1.19^ab^	0.7^ab^	0.46^ab^	1.18^ab^	1.07^b^	1.11^ab^	1.18^a^	0.52	8.19E–01	7.18E–01	1.03E–01
Parabacteroides	0.58^abc^	0.63^abc^	0.75^ab^	0.39^ab^	0.29^c^	0.45^c^	0.45^bc^	1.10^a^	0.65	5.14E–02	2.21E–02	2.37E–01
Rikenella	0.55^a^	1.01^a^	0.74^a^	0.17^b^	0.79^a^	1.72^a^	0.88^a^	0.13^b^	0.70	3.73E–06	4.55E–01	7.72E–01
Rikenellaceae RC9 gut group	0.40^ab^	0.33^a^	0.2^ab^	0.2^a^	0.51^a^	0.32^ab^	0.20^b^	0.46^a^	0.25	1.55E–02	4.19E–01	6.12E–01
Romboutsia	1.39^a^	0.07^b^	0.00^d^	0.00^d^	1.42^a^	0.02^c^	0.00^d^	0.00^d^	0.37	8.77E–26	2.45E–03	1.18E–03
Roseburia	0.54^a^	0.11^bcd^	0.14^abc^	0.06^cd^	0.56^ab^	0.49^abc^	0.51^abc^	0.07^d^	0.40	1.23E–03	6.26E–01	1.87E–01
Ruminiclostridium	2.78^a^	1.44^bc^	1.26^abc^	0.64^c^	3.48^a^	3.35^a^	2.86^a^	1.92^ab^	1.28	7.14E–02	2.51E–03	3.77E–01
Ruminiclostridium 5	0.55^a^	0.39^ab^	0.24^ab^	0.12^bc^	0.64^a^	0.63^a^	0.46^a^	0.16^c^	0.19	7.74E–05	8.75E–01	1.79E–01
Ruminiclostridium 9	2.68^abc^	1.99^bcd^	1.40^bcd^	0.77^d^	4.52^a^	2.85^abc^	3.27^ab^	1.58^cd^	1.13	2.73E–03	2.45E–02	8.46E–01
Ruminococcaceae NK4A214 group	0.12^ab^	0.10^ab^	0.07^ab^	0.07^a^	0.02de	0.07^bc^	0.04^cd^	0.01e	0.05	2.38E–01	3.99E–07	2.19E–02
Ruminococcaceae UCG-003	0.61^c^	1.27^ab^	1.26^a^	0.67^ab^	1.25^b^	2.17^a^	1.46^ab^	1.11^ab^	0.79	6.42E–03	5.63E–01	3.53E–01
Ruminococcaceae UCG-004	0.18^a^	0.10^ab^	0.06^b^	0.02^c^	0.22^ab^	0.15^ab^	0.17^ab^	0.00^d^	0.05	3.30E–15	5.73E–03	4.57E–08
Ruminococcaceae UCG-009	0.10^a^	0.06^ab^	0.05^ab^	0.02^b^	0.13^a^	0.12^a^	0.07^ab^	0.10^a^	0.06	2.08E–01	7.50E–02	3.38E–01
Ruminococcaceae UCG-014	0.37^ab^	0.48^a^	0.07^bc^	0.06^c^	0.13^bc^	0.14^bc^	0.02^d^	0.02^d^	0.26	1.16E–05	1.07E–05	6.35E–01
Ruminococcus 1	0.50^abc^	0.21^bc^	0.38^a^	0.23^ab^	0.18^c^	0.41^abc^	0.33^abc^	0.19^c^	0.40	2.55E–01	7.96E–02	1.22E–01
Subdoligranulum	0.13^a^	0.00de	0.04^bc^	0.04^ab^	0.03^cd^	0.00e	0.00e	0.00e	0.05	1.28E–04	1.15E–08	1.84E–02
Turicibacter	4.21^a^	1.44^b^	1.17^b^	0.00^d^	2.66^b^	0.00^d^	0.05^c^	0.00^d^	1.01	9.13E–23	1.81E–15	4.06E–14
Tyzzerella	0.43^bcd^	0.29^d^	0.22^cd^	0.18^bcd^	0.77^abc^	0.45^bcd^	0.73^ab^	0.81^a^	0.32	8.95E–02	1.04E–03	3.22E–01

^1^Data was anayzed by a two-way ANOVA.

Groups with different letters are significantly differernt, *p* < 0.05.

**Table 2:** Effect of diet and *C. rodentium* infection on various genera with relative abudance above 0.1% from feces obtained from uninfected and infected mice at day 6 post-infection^1^.

**Treatment**	**Uninfected**				**Infected**				**Pooled SEM**			Treatment *infection
	**0%**	**2%**	**5%**	**10%**	**0%**	**2%**	**5%**	**10%**		**Treatment**	**Infection**	
[Eubacterium] coprostanoligenes group	0.36^a^	0.02^b^	0.00^c^	0.00^c^	0.07^ab^	0.00^c^	0.00^c^	0.00^c^	0.27	6.09E–10	8.25E–03	4.56E–02
Akkermansia	10.16^b^	14.96^a^	14.52^a^	11.11^a^	19.74^a^	14.07^ab^	14.56^a^	11.91^a^	3.92	4.79E–02	1.00E–01	2.66E–01
Alistipes	9.61^abc^	9.44^ab^	8.87^a^	3.18^c^	16.35^a^	9.35^ab^	7.93^a^	2.96^c^	3.77	5.36E–03	2.77E–01	6.00E–01
Azospirillum sp. 47_25	0.31^c^	0.38^bc^	0.52^ab^	0.95^a^	0.93^ab^	0.51^abc^	0.91^a^	0.45^abc^	0.58	6.76E–02	1.99E–01	3.54E–02
Bacteroides	0.54^c^	0.36^c^	2.55^b^	4.41^ab^	1.10^c^	0.35^c^	6.01^a^	7.96^a^	3.48	3.55E–10	2.38E–02	7.87E–01
Bifidobacterium	0.34^b^	3.06^a^	1.83^a^	2.94^a^	0.14^b^	2.06^a^	2.88^a^	1.80^a^	1.54	9.72E–09	2.06E–01	2.30E–01
Bilophila	1.62^a^	0.53^bc^	0.51^abc^	0.23^cd^	0.98^ab^	0.72^abc^	0.27^bc^	0.10^d^	0.61	3.11E–04	2.39E–01	1.98E–01
Blautia	1.97^a^	0.93^a^	0.45^ab^	0.23^b^	0.80^ab^	1.03^ab^	0.38^ab^	0.07^c^	0.92	7.44E–04	9.94E–02	4.07E–01
Catenibacterium	0.14^d^	0.58^ab^	0.2^cd^	0.60^a^	0.25^cd^	0.13^d^	0.29^bc^	0.75^a^	0.25	1.09E–06	8.85E–01	3.88E–03
Citrobacter	0.00^c^	0.00^c^	0.00^c^	0.00^c^	5.56^b^	3.30^b^	7.45^a^	11.29^a^	3.57	1.51E–02	1.16E–22	3.67E–02
Clostridium sensu stricto 1	17.17^a^	8.49^a^	0.01^bc^	0.01^bc^	12.92^a^	0.01^b^	0.00^c^	0.00^bc^	4.50	1.59E–18	1.21E–08	1.67E–08
Desulfovibrio	0.65^bcd^	0.32^bcd^	0.52^abc^	0.54^ab^	0.34^d^	0.32^cd^	0.22^cd^	0.66^a^	0.42	8.97E–04	2.80E–01	2.51E–01
Dubosiella	0.03^d^	0.11^abc^	0.03^cd^	0.19^ab^	0.04^d^	0.06^cd^	0.09^bc^	0.23^a^	0.08	3.90E–06	4.33E–01	6.59E–02
Erwinia	0.00^d^	0.00^d^	0.00^d^	0.00^d^	0.23^c^	0.18^c^	0.32^b^	0.46^a^	0.15	9.05E–03	1.90E–19	9.05E–03
Faecalibaculum	9.75^b^	28.25^a^	22.03^a^	30.66^a^	5.95^b^	31.30^a^	26.05^a^	31.90^a^	10.99	2.45E–06	9.15E–01	5.41E–01
GCA-900066575	0.62^a^	0.23^ab^	0.21^ab^	0.11^b^	0.62^ab^	0.27^ab^	0.19^ab^	0.35^ab^	0.40	3.01E–01	6.03E–01	2.91E–01
Lachnoclostridium	1.47^a^	0.44^bc^	0.29^bc^	0.17^c^	0.84^ab^	1.00^a^	0.23^c^	0.22^bc^	0.52	6.32E–05	5.53E–01	3.91E–02
Lachnospiraceae NK4A136 group	2.79^d^	12.45^c^	31.61^a^	34.02^a^	3.69^d^	13.77^c^	14.48^bc^	15.89^b^	9.24	1.27E–16	9.79E–03	1.16E–03
Lachnospiraceae UCG-006	0.17^a^	0.05^ab^	0.05^ab^	0.06^ab^	0.14^a^	0.05^ab^	0.03^b^	0.0^ab^	0.09	5.50E–02	4.85E–01	9.91E–01
Lactobacillus	12.69^a^	4.16^ab^	2.29^b^	0.41^c^	4.52^ab^	4.78^ab^	5.71^ab^	1.21^b^	5.42	3.34E–03	3.90E–01	1.02E–01
Mucispirillum	2.55^ab^	0.34^b^	0.60^ab^	0.55^ab^	0.73^b^	0.37^b^	0.54^ab^	1.01^a^	1.86	1.37E–01	8.61E–01	8.06E–02
Odoribacter	0.6^bc^	0.58^ab^	0.56^ab^	0.50^ab^	0.83^ab^	1.22^a^	0.64^ab^	0.08^c^	0.62	4.80E–02	9.44E–01	9.95E–03
Oscillibacter	1.01^a^	0.50^ab^	0.46^abc^	0.29^bc^	0.57^abc^	0.60^ab^	0.30^bc^	0.21^c^	0.43	7.94E–02	7.98E–02	4.97E–01
Parabacteroides	0.88^cde^	0.69^cde^	0.99^bcd^	1.28^ab^	0.64^de^	0.44^e^	1.07^bc^	2.37^a^	0.81	2.32E–05	8.52E–01	1.39E–01
Rikenella	0.17^a^	0.07^a^	0.09^a^	0.01^b^	0.19^a^	0.16^a^	0.11^a^	0.01^b^	0.06	4.07E–08	1.64E–01	7.38E–01
Rikenellaceae RC9 gut group	0.84^abc^	0.42^bcd^	0.32^cd^	0.73^a^	0.92^ab^	0.80^a^	0.23^d^	0.56^a^	0.31	3.92E–04	3.43E–01	2.98E–01
Romboutsia	0.97^a^	0.01^b^	0.00^c^	0.00^c^	1.02^a^	0.00^c^	0.00^c^	0.00^c^	0.30	6.83E–24	3.26E–03	1.08E–03
Roseburia	0.29^a^	0.03^abc^	0.02^bc^	0.01^c^	0.07^ab^	0.03^abc^	0.02^bc^	0.01^c^	0.16	5.67E–04	4.61E–01	7.27E–01
Ruminiclostridium	1.79^a^	0.60^abc^	0.73^ab^	0.36^bc^	1.15^ab^	1.04^a^	0.35^c^	0.33^c^	0.65	3.98E–03	3.25E–01	8.54E–02
Ruminiclostridium 5	0.37^a^	0.15^ab^	0.12^ab^	0.06^b^	0.15^ab^	0.1^ab^	0.07^b^	0.06^b^	0.15	6.29E–02	2.07E–01	5.71E–01
Ruminiclostridium 9	1.88^a^	1.25^a^	0.68^bc^	0.36^d^	1.59^a^	1.27^ab^	0.42^cd^	0.25^d^	0.59	1.53E–08	8.60E–02	8.92E–01
Ruminococcaceae NK4A214 group	0.27^a^	0.13^ab^	0.11^ab^	0.12^a^	0.14^ab^	0.06^bc^	0.05^c^	0.02^c^	0.08	1.07E–01	9.19E–05	1.81E–01
Ruminococcaceae UCG-003	0.67^ab^	0.79^ab^	0.65^ab^	0.24^b^	0.99^ab^	1.00^a^	0.68^a^	0.45^ab^	0.61	6.24E–02	2.30E–01	9.92E–01
Ruminococcaceae UCG-004	0.13^a^	0.04^a^	0.01^b^	0.00^bc^	0.08^a^	0.03^a^	0.01^bc^	0.00^c^	0.06	1.22E–07	4.31E–01	9.91E–01
Ruminococcaceae UCG-014	0.80^abc^	1.49^a^	0.07^c^	0.70^ab^	1.65^a^	0.22^bc^	0.11^bc^	0.88^ab^	0.93	6.46E–03	8.38E–01	1.20E–01
Ruminococcus 1	1.47^a^	0.17^a^	0.91^a^	0.42^a^	0.24^a^	0.27^a^	0.42^a^	0.22^a^	1.22	3.31E–01	1.76E–01	5.87E–01
Subdoligranulum	0.21^a^	0.01^bc^	0.03^abc^	0.02^bc^	0.04^ab^	0.01^bc^	0.00^c^	0.02^bc^	0.09	2.37E–02	6.46E–02	6.02E–01
Turicibacter	4.49^a^	0.90^b^	0.53^b^	0.00^c^	1.29^b^	0.00^c^	0.00^c^	0.00^c^	1.62	3.80E–15	1.74E–12	8.85E–09
Tyzzerella	0.29^a^	0.11^a^	0.15^a^	0.11^a^	0.21^a^	0.17^a^	0.14^a^	0.18^a^	0.17	3.00E–01	7.43E–01	6.07E–01

^1^Data was anayzed by a two-way ANOVA.

Groups with different letters are significantly different, *p* < 0.05.

**Figure 6:**
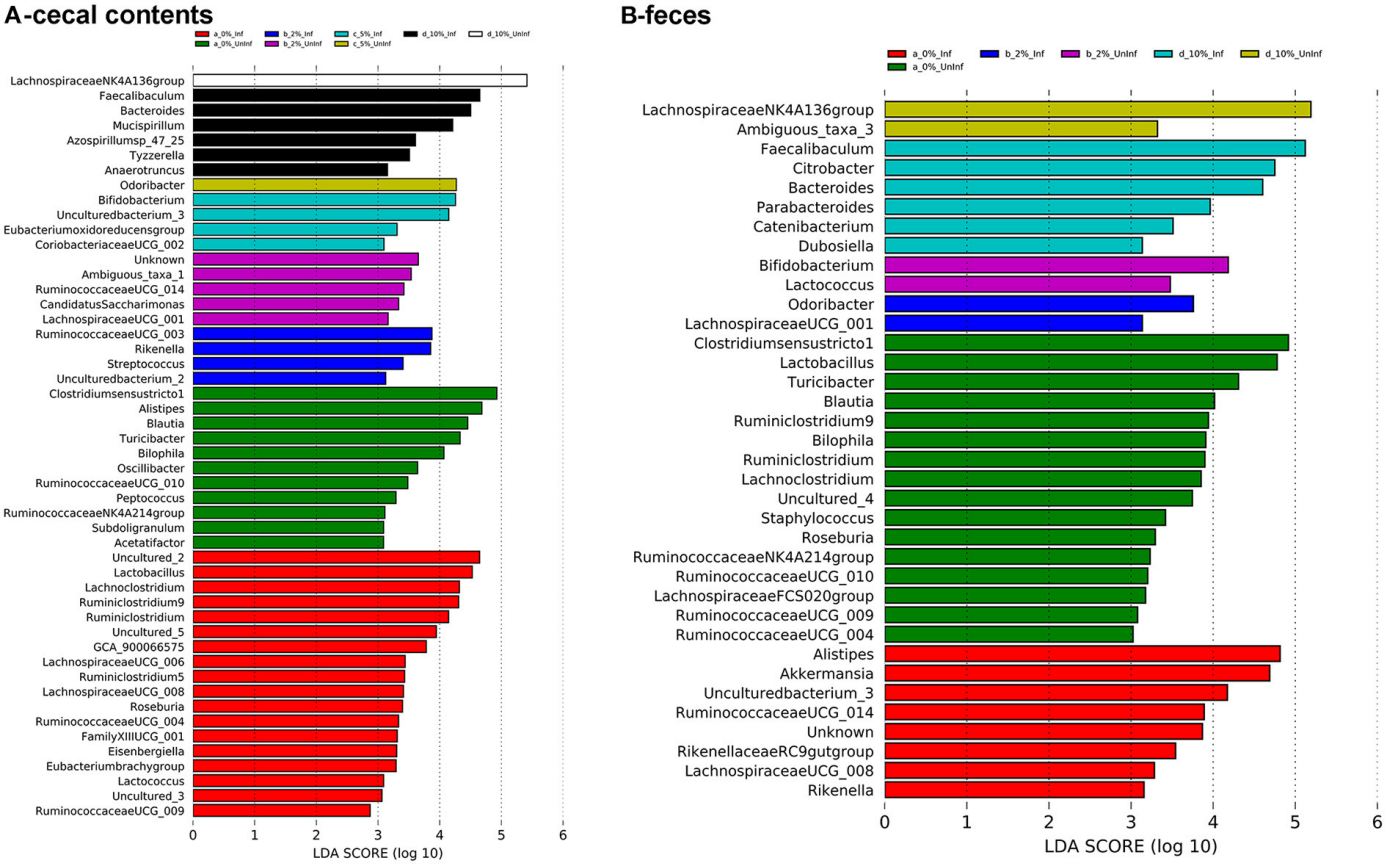
LEfSe plots identify discriminating genera in uninfected and infected mice. LEfSe plots were generated from16S sequences data from uninfected and infected mice fed different levels of dietary RPS and identified various genera that were discriminant for the various treatments. **(A)** Cecal contents, **(B)** Feces. An LDA value >2 is considered significant, *n* = 4–5/group.

To identify bacterial genera that discriminate between consumption of different levels of dietary RPS and infection status, LEfSe plots were generated for D6 post-infection fecal and D12 post-infection cecal samples (Figure 6A). The all-group LEfSe comparison of D12 cecal samples showed that *Lachnospiraceae NK4136 group* was most discriminating for uninfected mice fed the 10% RPS diet while *Odoribacter, Ruminococcaceae UCG-014*, and *Clostridium senso stricto 1* were most discriminating for uninfected mice fed the 5, 2, and 0% RPS diets, respectively. *Alistipes* and *Balautia* and *Turcibacter* were highly discriminating for uninfected mice fed the 0% RPS diet. LEfSe analysis of infected mice fed the 10% RPS diet identified *Faecalibaculum, Bacteroides*, and *Mucispirillum* as most discriminant. For infected mice fed the 5% RPS diet *Bifidobacterium* was most discriminant while *Ruminococcaceae UCG-003* and *Rikenella* were most discriminant for infected mice fed the 2% RPS diet. Several genera were discriminant for infected mice fed the 0% RPS diet including *Lactobacillus, Lachnoclostridium and Ruminiclostridium 9*.

LEfSe analysis of D6 post-infection fecal samples (Figure 6B) again identified *Lachnospiraceae NKA136 group* as most discriminant for uninfected mice fed the 10% RPS diet while *Faecalibaculum* and *Bacteroides* again were discriminant for infected mice fed the 10% RPS diet. No discriminating genera were identified for the uninfected and infected 5% RPS groups. Fecal samples from uninfected mice fed the 0% RPS diet shared several discriminating genera with cecal samples including *Clostridium senso stricto 1, Blautia, Turicibacter*, and *Bilophia*. The common characteristic of these discriminating genera in the LEfSe plots for mice fed the 0% RPS diet is that their relative abundance all decrease with increasing dietary RPS (Table 2). No common discriminating genera were shared between D6 fecal and D12 cecal uninfected or infected groups fed the 2% RPS diet with *Bifidobactium* being most highly discriminant in uninfected mice and *Odoribacter* in infected mice. Nor was there overlapping discriminant genera between D6 fecal and D12 cecal samples from infected mice fed the 0% RPS diet with *Alistipes* and *Akkermansia* being most discriminant for D6 fecal samples (Figures 6A, B).

### Effect of RPS and Cr infection on gene expression in the cecum and distal colon

To explore potential mechanisms that could be associated with the increased colonization of *Cr* observed in animals fed the 10% RPS diet, we conducted RNASeq analysis on the cecum and DC of infected or control animals that were fed a control diet 2, 5, or 10% RPS. The eight groups of animals will be referred to in the text as follows; uninfected animals fed the control diet, 2, 5, or 10% RPS will be referred to as 0U, 2U, 5U and 10U. Infected animals fed the control diet, 2, 5, or 10% RPS will be referred to as 0I, 2I, 5I and 10I. Comparisons will be referred to by an underscore (_). All pairwise comparisons for cecum and DC are found in Supplementary Tables 2, 3, respectively.

Differentially expressed genes in cecum formed three distinct clusters by PCA (Supplementary Figure 6B) and two in DC (Supplementary Figure 6D). This is due to the overwhelming effect of infection. In the cecum, the proportion of variance was greatest for infection (48%), the residual (39%), the interaction of infection and RPS treatment (9%) and RPS (3.7%) treatment (Supplementary Figure 6A). Similarly, in DC, the proportion of variance was greatest for infection (49%), the residual (44%), the interaction of infection and RPS treatment (4.9%) and RPS (2.7%) treatment (Supplementary Figure 6C). This is in contrast to variance analysis for the cecal and fecal microbiota, which were significantly affected by diet (Supplementary Figure 4).

In the cecum, the top three number of DEGs occurred in the comparisons with 10I_0U, 10I_10U and 10I_2U (Table 3). The ratio of down to upregulated genes in these comparisons was 1.2. In the DC, the top two number of DEGs occurred in the comparisons with 10I_10U and 10I_0U (Table 4). The ratio of down to upregulated genes in these comparisons was 1.0 and 1.6. This indicates that the increased number of differentially expressed genes, seen in infected animals fed the 10%RPS, derived mostly from downregulated genes suggesting that 10% RPS has a general suppressive effect on gene expression in the cecum and DC of infected animals.

**Table 3:** Top 20 differentially expressed genes in the infected cecum.

**Downregulated**											
**0I_0U**			**2I_0U**			**5I_0U**			**10I_0U**		
**Gene**	**FC**	***p* (adj)**	**Gene**	**FC**	***p* (adj)**	**Gene**	**FC**	***p* (adj)**	**Gene**	**FC**	***p* (adj)**
Wdr17	−53.6	4.13E-03	Cyp3a44	−132.7	2.94E-02	Fgf15	−25584041.7	4.91E-14	Trpv6	−5803.4	7.92E-31
Cd209c	−30.9	1.56E-02	Cfap57	−51.8	4.32E-03	2010106E10Rik	−17802706.4	9.16E-13	Cyp24a1	−1343.6	7.44E-15
Lrrc7	−25.6	1.59E-02	Myh7	−45.2	1.90E-02	Defa24	−8640418.1	2.92E-10	Acot12	−267.7	4.58E-16
Slitrk4	−21.0	3.12E-02	Mdfic2	−44.2	3.00E-02	Defa22	−5571387.3	2.68E-08	Cyp2c69	−180.5	1.23E-13
Ncr1	−19.9	2.16E-02	Gm21190	−39.1	9.85E-05	Defa30	−4361105.7	9.58E-08	Cyp4b1	−158.1	2.46E-41
Cyp2c69	−17.0	4.97E-06	Nr0b2	−36.6	1.45E-02	Defa21	−4127103.0	4.10E-07	Ugt8a	−154.0	1.04E-25
Kel	−10.0	4.16E-02	Cyp24a1	−32.8	1.38E-05	Defa38	−3566779.8	2.26E-07	9330182O14Rik	−144.4	1.84E-12
Mug2	−9.7	1.24E-02	Cyp2c69	−31.7	6.31E-08	Defa39	−552037.7	1.46E-04	Llcfc1	−120.3	1.07E-06
Slc4a5	−9.6	1.15E-02	Tmprss11e	−31.6	3.58E-02	Apoa4	−299.3	3.46E-06	Tgm3	−81.8	1.27E-07
Cyp2a12	−9.4	3.12E-02	Acot5	−31.6	1.24E-03	Apoa1	−200.2	1.68E-04	Cdh20	−76.8	9.71E-08
Cdh20	−7.6	1.86E-02	Cyp4b1	−31.3	1.90E-17	Cyp3a25	−180.9	2.31E-02	Mptx1	−76.0	1.31E-11
Tmem52b	−7.0	2.42E-02	Gm21083	−31.3	2.81E-03	Cyp2c69	−173.1	5.60E-11	1700057G04Rik	−74.1	2.56E-09
Angptl1	−6.5	1.55E-02	Acot12	−31.1	1.28E-13	Cyp3a44	−137.4	2.49E-02	Pbld1	−69.0	2.18E-33
Dscaml1	−6.1	9.92E-03	Plpp4	−28.5	4.17E-02	Pdzk1	−80.7	1.79E-02	Cyp2c55	−68.5	8.11E-40
Phyhip	−5.9	3.66E-02	Ugt8a	−28.3	4.11E-12	G6pc	−80.7	1.22E-03	AU018091	−68.1	1.39E-05
A730046J19Rik	−5.8	8.15E-03	Trpv6	−25.4	1.34E-06	Llcfc1	−77.3	7.46E-05	C9	−59.2	3.67E-06
Depp1	−5.5	4.07E-02	Cyp4a10	−24.9	4.25E-02	AU018091	−65.3	1.13E-04	BC035947	−53.7	7.25E-04
Lingo1	−5.3	5.23E-03	Tmprss11a	−22.9	1.30E-02	Cyp4b1	−63.8	2.80E-25	Trpv5	−53.6	3.41E-05
Dbp	−5.0	2.14E-02	Il1rapl1	−22.5	2.52E-02	Olfm4	−58.9	1.44E-02	Elovl3	−53.5	2.75E-04
Ano5	−5.0	1.09E-02	4933407L21Rik	−22.2	2.86E-02	Cyp3a11	−56.4	3.87E-02	Mettl7a3	−53.1	2.71E-06
**Upregulated**											
**0I_0U**			**2I_0U**			**5I_0U**			**10I_0U**		
**Gene**	**FC**	***p*** **(adj)**	**Gene**	**FC**	***p*** **(adj)**	**Gene**	**FC**	***p*** **(adj)**	**Gene**	**FC**	***p*** **(adj)**
Ighe	36.7	4.53E-03	Gzmk	68.4	4.86E-11	Tgtp1	69.3	3.79E-07	Ighv7-4	76.8	4.78E-03
Gbp10	37.2	2.62E-03	Ighv1-74	73.0	1.11E-03	Igkv8-30	74.7	3.81E-13	Gzmk	77.0	2.47E-05
Serpina10	40.2	3.08E-04	Ighv9-2	75.7	9.25E-07	Gzmk	75.1	9.25E-05	Il36g	78.4	5.76E-06
Igkv8-28	42.0	1.68E-03	Igkv13-85	78.1	4.93E-02	Ighv1-54	85.7	2.65E-08	Tnfsf4	79.3	5.75E-06
Gzma	43.3	2.07E-06	Igkv9-129	83.1	1.07E-05	Gbp10	86.4	1.14E-05	Igkv3-1	84.7	1.45E-04
Gm8369	50.1	2.86E-04	Rnase2b	83.9	1.71E-05	Nos2	86.8	1.74E-14	Gzmg	84.8	3.68E-02
Ifng	57.7	5.46E-04	Ifng	84.7	1.76E-05	Ighv1-61	95.8	4.81E-04	Igkv4-79	92.4	1.91E-04
Rnase2b	62.8	2.72E-04	Gbp10	85.7	1.11E-13	Ighv14-4	105.7	1.61E-03	Ighv1-59	96.1	1.01E-11
Igkv8-30	67.8	5.72E-10	Igkv8-30	90.8	6.13E-11	Gzma	116.9	2.44E-12	Lin28a	105.3	3.57E-06
Saxo1	70.1	3.45E-05	Gzma	95.0	6.38E-07	Gm4841	132.7	3.66E-12	Ighv1-63	113.5	8.05E-12
Ighv5-12	75.6	2.78E-05	Saxo1	139.8	4.78E-07	Saxo1	136.4	3.95E-08	Saxo1	157.9	2.16E-09
Ighv1-63	93.4	6.73E-08	Serpina10	141.7	2.68E-08	F830016B08Rik	160.0	1.14E-07	Nos2	170.0	1.56E-21
Igkv3-1	98.8	1.64E-03	Ighv1-61	142.5	1.69E-04	Ifng	160.3	3.90E-07	Serpina10	183.1	3.63E-10
Igkv4-79	105.3	2.23E-03	Ighv1-5	179.1	7.61E-05	Serpina10	167.1	5.32E-09	Ly6f	197.1	5.75E-09
Ly6f	125.2	1.07E-05	Ighv14-4	184.0	4.56E-04	Ighv8-5	179.8	3.10E-02	Gm4841	233.6	8.35E-17
Igkv12-89	137.7	4.45E-03	Igkv12-89	189.9	4.48E-04	Ly6f	251.9	8.21E-09	Tgtp1	249.1	1.79E-12
Ighv7-4	168.0	8.43E-03	Igkv4-79	257.9	2.23E-05	Igkv12-89	317.8	7.60E-05	Gbp10	264.6	2.99E-09
Ighv14-4	301.6	7.33E-04	Ighv1-63	401.6	4.41E-16	Igkv4-79	318.4	7.11E-06	Ifng	276.8	1.96E-09
Ighv1-59	405.9	6.41E-14	Ighv1-59	558.7	8.70E-19	Ighv1-59	655.3	3.70E-20	F830016B08Rik	336.0	9.11E-11
Ighv2-4	5454179.4	1.11E-06	Ighv2-4	563212.3	2.87E-06	Ighv1-63	719.8	1.33E-19	Ighv2-4	433665.1	4.48E-07

**Table 4:** Top 20 differentially expressed genes in the infected distal colon.

**Downregulated**											
**0I_0U**			**2I_0U**			**5I_0U**			**10I_0U**		
**Gene**	**FC**	***p* (adj)**	**Gene**	**FC**	***p* (adj)**	**Gene**	**FC**	***p* (adj)**	**Gene**	**FC**	***p* (adj)**
Methig1	−69.9	9.71E-04	Mettl7a2	−133.8	4.62E-05	Oaz3	−189.2	5.41E-07	Scgb1b3	−157.4	1.54E-11
Fxyd4	−48.7	1.06E-06	Methig1	−122.9	1.23E-05	Mettl7a2	−126.1	5.80E-05	Mptx1	−153.8	1.32E-16
Slc28a2b	−46.6	1.22E-03	Wnt8b	−122.5	7.25E-05	Mettl7a3	−120.0	2.21E-06	Mettl7a2	−151.5	6.44E-06
Mettl7a2	−43.3	6.92E-03	Cyp2c69	−95.8	1.59E-11	Fxyd4	−104.8	6.61E-10	Methig1	−139.1	1.24E-06
Prg3	−40.0	4.71E-02	Oaz3	−89.6	1.77E-05	Clcnkb	−77.4	1.20E-05	Wnt8b	−138.7	1.09E-05
Grin2b	−37.8	2.22E-03	Clcnkb	−82.2	8.73E-06	Wnt8b	−77.4	3.57E-04	Slc17a2	−127.2	3.45E-10
Cyp2c67	−35.3	7.67E-03	Klk13	−68.7	9.32E-06	Slc30a10	−60.4	4.48E-14	Ighv1-11	−119.4	1.20E-04
Sytl5	−32.2	2.65E-03	Slc30a10	−63.0	1.96E-14	A630010A05Rik	−57.5	7.02E-06	Igkv9-123	−100.0	1.96E-08
Slc35f4	−31.8	9.49E-03	Mettl7a3	−57.5	6.57E-05	Mptx1	−52.3	1.06E-09	Clcnkb	−93.0	6.98E-07
Lrrc74b	−30.3	8.29E-03	Gm21190	−56.7	3.36E-08	Klhdc7b	−49.6	7.58E-05	Slc30a10	−85.5	4.62E-18
Htr1d	−30.2	2.21E-02	Il25	−54.8	6.92E-06	Wfdc6b	−48.1	4.31E-03	Glycam1	−83.4	8.08E-08
Lhfpl1	−28.3	9.51E-03	Mptx1	−53.7	6.94E-10	Atp13a4	−43.9	5.11E-05	Grin2b	−75.2	3.11E-06
Ly6g6g	−28.0	2.17E-02	Wfdc8	−42.1	3.54E-05	Glycam1	−42.6	1.81E-05	Adamts18	−73.4	3.43E-13
Clcnkb	−27.4	3.96E-03	Tgm3	−41.6	5.72E-08	Wfdc8	−39.7	4.80E-05	Igfn1	−69.7	7.76E-08
Tbx18	−26.5	3.94E-02	Wfdc16	−40.8	3.50E-04	Wfdc16	−38.4	4.33E-04	Ighv1-4	−68.6	1.05E-04
Trp63	−24.4	3.41E-02	9330182O14Rik	−40.7	8.46E-04	Gm3164	−36.5	5.11E-03	Dipk1c	−61.5	2.32E-06
Mfsd13b	−23.2	4.86E-04	Pgpep1l	−38.7	4.57E-04	Cyp2c40	−34.7	5.17E-05	Gm21190	−60.4	5.82E-10
Cibar2	−23.1	5.39E-03	Cyp2c67	−37.7	1.50E-03	Apoc3	−32.0	3.14E-03	Sdr16c6	−58.9	1.35E-03
Spata3	−20.9	1.32E-02	Cyp2c40	−36.8	3.77E-05	Slc28a2b	−31.4	7.61E-04	Smim38	−58.8	1.52E-03
Hapln3	−20.4	1.29E-02	Hmx3	−34.0	1.66E-04	Sox1	−30.6	3.73E-04	Fxyd4	−54.6	1.30E-09
**Upregulated**											
**0I_0U**			**2I_0U**			**5I_0U**			**10I_0U**		
**Gene**	**FC**	***p*** **(adj)**	**Gene**	**FC**	***p*** **(adj)**	**Gene**	**FC**	***p*** **(adj)**	**Gene**	**FC**	***p*** **(adj)**
Nos2	60.9	6.14E-35	Ccl1	125.6	5.39E-08	Gzmk	75.4	5.98E-06	Serpinb2	123.4	2.49E-04
Olfr1512	63.9	4.16E-06	Clec4e	153.2	2.82E-20	Il22	86.5	5.32E-05	Reg3d	124.0	1.02E-02
2310034C09Rik	65.0	5.90E-10	Nos2	175.2	2.17E-63	Olfr1512	98.2	3.07E-08	Il36g	128.9	6.68E-06
Ccl1	72.3	1.17E-05	Sirpb1c	186.2	2.30E-10	Nos2	113.2	5.89E-53	Clec4e	133.7	2.57E-20
Gml	75.5	4.69E-10	Gml	201.7	1.33E-16	Clec4e	116.3	4.57E-18	Gml	138.7	2.97E-15
Krt6b	75.9	4.18E-06	Krt6a	210.0	8.95E-05	Sirpb1c	129.3	4.84E-09	Krt6b	151.6	1.55E-09
Sirpb1c	77.6	1.35E-06	Il36g	232.8	1.10E-06	Gml	129.4	5.36E-14	Chil3	162.6	7.74E-07
S100a9	94.5	6.85E-05	Reg3g	275.6	4.09E-14	Krt6b	154.1	4.47E-09	Sirpb1c	243.8	7.17E-12
Mcpt9	100.2	2.48E-05	Krt6b	317.7	1.16E-11	Cxcl3	192.6	1.11E-07	Cxcl5	271.5	3.51E-16
Reg3g	105.3	2.12E-08	Reg3b	339.1	1.36E-11	Cxcl5	282.7	6.51E-15	Reg3g	282.3	1.19E-15
Cxcl3	121.1	1.09E-05	Cxcl3	436.0	5.70E-10	Prss22	283.2	3.11E-08	Gml2	288.3	6.38E-11
Cxcl5	127.4	1.38E-09	Gml2	476.9	4.15E-12	Gml2	294.6	2.34E-10	Cxcl3	293.8	2.20E-09
Reg3b	140.1	3.27E-07	Prss22	553.7	4.28E-10	Reg3g	319.0	1.05E-14	Reg3b	368.8	4.34E-13
Gml2	165.3	1.44E-07	Cxcl5	572.2	1.04E-18	S100a9	325.3	1.38E-08	S100a8	445.8	4.55E-11
Prss22	169.0	4.70E-06	Clca4b	768.1	1.46E-92	S100a8	346.9	1.66E-09	Prss22	457.5	3.66E-10
S100a8	180.2	1.20E-06	Sprr2h	968.0	5.25E-27	Reg3b	375.4	7.07E-12	Sprr2h	506.6	4.89E-24
Sprr2h	264.5	1.01E-15	S100a9	979.6	8.83E-12	Clca4b	553.8	1.70E-83	Clca4b	533.7	5.21E-90
Clca4b	282.0	1.13E-58	Reg2	1034.0	3.12E-04	Sprr2h	620.8	1.20E-23	S100a9	609.9	3.92E-11
Reg2	758.6	2.54E-03	S100a8	1105.2	3.05E-13	Reg2	781.4	5.47E-04	Reg2	1898.8	2.85E-05
Reg3a	1022.6	3.71E-05	Reg3a	2025.8	3.86E-07	Reg3a	948.7	5.66E-06	Reg3a	2870.8	2.42E-08

Because of space limitations, we will focus on three sets of comparisons when discussing genes and pathways unless otherwise indicated. In the first, the fold-change from the 2U_0U, 5U_0U or 10U_0U comparisons will be compared to changes in the 0I_0U comparison to elucidate the magnitude of the changes due to differing levels of dietary RPS in uninfected mice compared to those induced by *Cr* infection in the absence of dietary RPS. In the second, changes in the 0I_0U, 2I_0U, 5I_0U or 10I_0U comparisons will be compared to the changes in the 0I_0U comparison to elucidate the magnitude of changes due to diet and infection compared to those induced by *Cr* infection in the absence of dietary RPS. In the third, changes in the 2I_0I, 5I_0I or 10I_I comparisons will be compared directly to each other to elucidate how dietary RPS is affecting gene expression due to infection.

The number of genes that were up- or downregulated >1.5 fold at an FDR adjusted *p* < 0.05 in 2U_0U, 5U_0U or 10I_0U groups vs. 0I_0 is shown in Supplementary Table 2, Supplementary Figure 7. In the cecum (Supplementary Figure 7A), 1, 20 and 323 genes were upregulated by 2U_0U, 5U_0U, 5U_0U or 10U_0U vs. 0I_0U, respectively. Zero, 25 and 257 genes were downregulated by 2U_0U, 5U_0U, 5U_0U or 10U_0U vs. 0I_0U, respectively (Supplementary Figure 7B). Venn Analysis revealed one upregulated gene exclusively in the 2U_0 comparison, two genes exclusively in the 5U_0U comparison and 305 upregulated genes in the 10U_0U comparison (Supplementary Table 2; Supplementary Figure 7A).

In DC, 17, 152 and 379 genes were upregulated by 2U_0U, 5U_0U, 5U_0U or 10U_0U vs. 0I_0U, respectively (Supplementary Table 3; Supplementary Figure 7C). Three, 99 and 168 genes were downregulated by 2U_0U, 5U_0U, 5U_0U or 10U_0U vs. 0I_0U, respectively (Supplementary Figure 7D). Venn Analysis revealed one upregulated gene exclusively in the 2U_0U comparison and two genes exclusively in the 5U_0U comparison (Supplementary Table 3; Supplementary Figure 7C).

**Figure 7:**
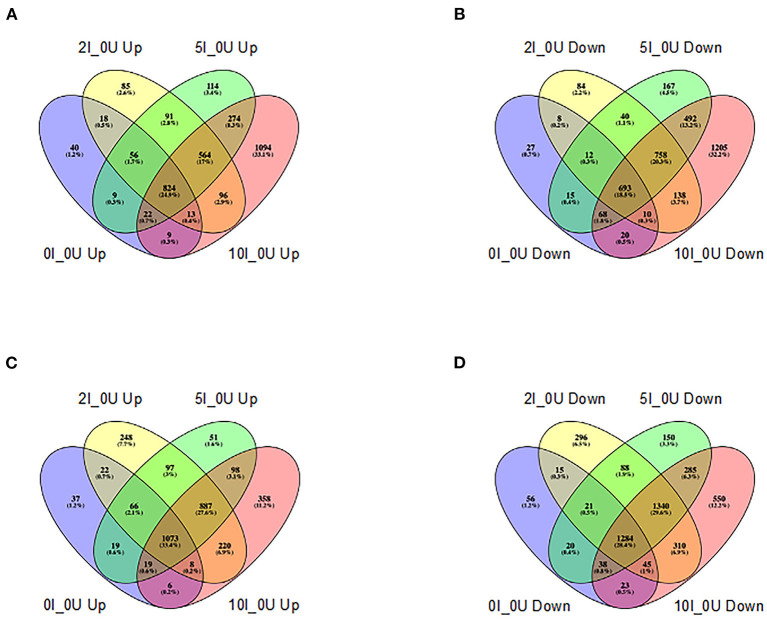
Venn analysis of differentially expressed genes in cecum and distal colon of *Cr*-infected mice. Differentially expressed genes (up-or downregulated) >1.5 fold at a FDR adjusted *p* < 0.05) in each tissue in animals fed 10% RPS, were analyzed by Venn analysis using the online tool Venny2.1 (https://bioinfogp.cnb.csic.es/tools/venny/). Genes that were upregulated **(A)** or downregulated **(B)** in cecum or upregulated **(C)** or downregulated **(D)** in DC. *n* = 4–5/group.

In the cecum, the number of genes that were up- or downregulated >1.5 fold at an FDR adjusted *p* < 0.05 in 0I_0, 2I_0, 5I_0 or 10I_0 groups vs. 0nf_0 is shown in Figures 7A, B. The number of genes that were commonly upregulated in 0I_0, 2I_0, 5I_0 and 10I_0 groups vs. was 824. The number of genes that were commonly downregulated in 0I_0, 2I_0, 5I_0 and 10I_0 groups vs. was 693. In DC, the number of genes that were commonly upregulated in the 0I_0U, 2I_0U, 5I_0U or 10I_0U groups was 1,073 (Figures 7C, D). 1,284 genes were commonly downregulated in 0I_0, 2I_0, “5I_0 and 10I_0 groups. A detailed Venn analysis of these comparisons is presented in Supplementary Tables 2, 3 for cecum and DC, respectively.

The top 20 down- or up- regulated genes (0I_0U, 2I_0U, 5I_0U or 10I_0U groups vs. 0I_0U) for cecum and DC are shown in Tables 3, 4, respectively. There was only one gene, Cyp2c69, that was among the top 20 downregulated genes in the cecum of RPS-fed Infected groups vs. 0U control animals. In the 5I_0U comparison, eight genes exhibited an extreme level (−552038 to −25,584,042 fold) of downregulation, among these were 6 alpha defensins (Defa21, Defa22, Defa24, Defa30, Defa38, Defa39) and Fgf15, a mouse gene that functionally orthologous to human FGF19. These genes were not significantly downregulated by any other RPS treatment. In cecum, seven upregulated genes were found in common (Gbp10, Serpina1, Ifng, Saxo1, Ighv1-59, Ighv1-63, Igkv4-79) in the infection vs. control comparisons. Except for Saxo1, these genes progressively increased in expression and significance with increasing dietary RPS concentrations. Only one gene, Gzmk, was exclusively found in the top 20 genes of RPS-treated mice vs. 0U control.

In DC, only 2 genes, Mettl7a2, Clcnkb, were found in the top 20 list of all downregulated genes. The most highly downregulated gene in the DC was Oaz3 (−189.2 fold) found in the 5I_0U comparison. Similar to the cecum, (and except for Oaz3), there was a generalized increase in significance and fold change with increasing RPS content of the diet. One gene, Cuzd1, exhibited an extreme level of induction (5379899, 7921739, 5913556 and 12731645 fold). We consider these findings artifactual; therefore, it was omitted from the cell cycle comparisons and discussion. Fifteen genes were found in common, in the top 20 for all groups and of those, $\frac{3}{4}$ of them can be classified into common functional groups: four REG Family Genes (Reg2, Reg3a, Reg3b, Reg3g), two chemokines (Cxcl3, Cxcl5), two calprotectins (S100a8, S1009a) and two LU Superfamily members (Gml, Gml2). The average expression levels of the genes exhibited a pattern observed for a large number of genes with the highest induction in the 2I_0U comparison followed by the 10I_0U, 5I_0U and then 0I_0U level. We deemed this response as biphasic. Reg3a was the highest expressed gene in all groups and its expression increased in a biphasic manner (1,022.6, 2,025.8, 948.7 and 2,870.8 fold) in 0I_0U, 2I_0U, 5I _0U and 10I_0U comparisons, respectively.

**Figure 8:**
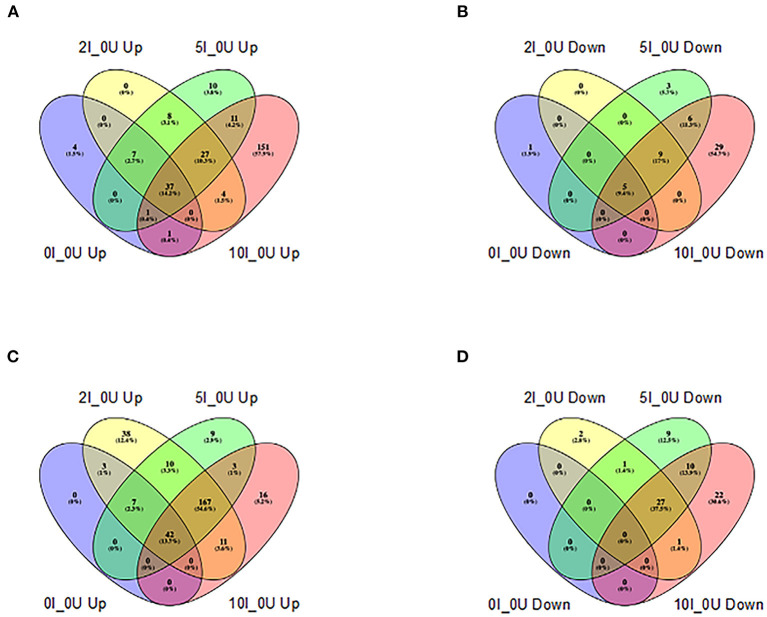
Venn analysis of shared REACTOME pathways of differentially expressed genes in cecum and distal colon of *Cr*-infected mice. Differentially expressed REACTOME pathways (up-or downregulated) >1.5 fold at a FDR adjusted *p* < 0.05) identified by DAVID analysis (https://david.ncifcrf.gov) in each tissue in animals fed 10% RPS, were analyzed by Venn analysis using the online tool Venny2.1 (https://bioinfogp.cnb.csic.es/tools/venny/). Genes that were upregulated **(A)** or downregulated **(B)** in cecum or upregulated **(C)** or downregulated **(D)** in DC are shown. *n* = 4–5/group.

Several gene pathways were parsed out for further analysis. Tables 5, 6 contain data for selected cytokines and markers of inflammation in the cecum and DC, respectively. Table 7 contains data for T Cell associated genes in the DC. All I_0U pairwise comparisons were analyzed for pathway enrichment using the online tool, DAVID and embedded Reactome database. Supplementary Tables 5, 6, contain the summary of all identified 0I_0U, 2I_0U, 5I_0U and 10I_0U Reactome pathways for the cecum and DC, respectively. Supplementary Table 7 contains a summary of I_0U pathway changes. Supplementary Table 8 contains data on cecum cell cycle pathways and Supplementary Table 9 contains data on individual cecum cell cycle genes. Similarly, Supplementary Table 10 contains data on DC cell cycle pathways and Supplementary Table 11 contains data on individual DC cell cycle genes. Supplementary Tables 12, 13 contains data on selected group of chemokines in the cecum and DC, respectively. A Venn analysis of the Reactome pathways is shown in Figure 8. Sixteen genes that were downregulated by RPS in DC are associated with vitamin A (VA) metabolism. This potential pathway was initially identified by REACTOME pathway analysis; however, numerous genes were missing and over half of them were associated with the pathway with little or no literature basis. Therefore, we used the PIN database to identify literature-based associations of genes with a defined role in VA metabolism. These genes were remapped into DAVID using KEGG and appear in Supplementary Figure 8.

**Table 5:** Selected differentially expressed genes in the cecum.

	**Statistical** **code**	**A**		**B**		**C**		**D**		**K**		**L**		**M**		**Q**		**R**		**S**	
		**0I_0U**		**2I_0U**		**5I_0U**		**10I_0U**		**2I_0I**		**5I_0I**		**10I_0I**		**5I_2I**		**10I_5I**		**10I_2I**	
**Categorization**	**Gene**	**FC**	***p* (adj)**	**FC**	***p* (adj)**	**FC**	***p* (adj)**	**FC**	***p* (adj)**	**FC**	***p* (adj)**	**FC**	***p* (adj)**	**FC**	***p* (adj)**	**FC**	***p* (adj)**	**FC**	***p* (adj)**	**FC**	***p* (adj)**
T Helper cell type																					
Th1-associated	Ifng	57.7	5.46E-04	83.9	1.71E-05	160.3	3.90E-07	276.8	1.96E-09	1.5	NS	2.8	NS	4.8	NS	1.9	NS	1.7	NS	3.3	NS
	Il12a	1.4	NS	−1.3	NS	−1.9	NS	−1.8	NS	−1.8	NS	−2.7	NS	−2.5	NS	−1.5	NS	1.1	NS	−1.4	NS
	Irf1	1.7	4.37E-05	2.1	2.42E-12	2.6	4.84E-21	2.5	5.07E-22	1.3	NS	1.6	2.14E-03	1.5	1.54E-04	1.3	NS	−1.0	NS	1.2	NS
	Stat1	2.3	9.71E-05	3.1	7.19E-11	4.3	2.76E-17	5.3	1.07E-25	1.4	NS	1.9	1.25E-02	2.4	1.89E-06	1.4	NS	1.2	NS	1.7	3.52E-03
	Irf8	1.7	4.20E-05	2.2	3.81E-12	2.8	2.28E-20	3.1	4.60E-28	1.3	NS	1.6	3.18E-03	1.8	4.86E-07	1.3	NS	1.1	NS	1.4	2.83E-03
	Ido1	3.5	1.35E-03	5.4	3.07E-07	10.4	2.17E-13	20.0	2.52E-24	1.5	NS	3.0	2.31E-02	5.7	1.35E-07	1.9	NS	1.9	NS	3.7	5.31E-05
	Nos2	17.9	3.45E-05	40.3	5.11E-10	86.8	1.74E-14	170.0	1.56E-21	2.2	NS	4.8	NS	9.5	2.91E-04	2.2	NS	2.0	NS	4.2	2.02E-02
TH17-associated	Il17a	2.1	NS	3.7	NS	3.0	NS	4.0	NS	1.8	NS	1.4	NS	1.9	NS	−1.2	NS	1.3	NS	1.1	NS
	Il17c	1.4	NS	2.8	NS	4.7	NS	6.3	3.77E-02	1.9	NS	3.3	NS	4.5	NS	1.7	NS	1.3	NS	2.3	NS
	Il22	25.5	NS	44.0	7.89E-03	36.6	1.04E-02	61.1	1.22E-03	1.7	NS	1.4	NS	2.4	NS	−1.2	NS	1.7	NS	1.4	NS
	Il22ra2	−1.8	NS	−3.4	2.14E-03	−3.1	4.73E-03	−33.6	2.25E-20	−1.9	NS	−1.7	NS	−19.0	2.69E-12	1.1	NS	−11.0	3.34E-08	−9.8	6.00E-08
Inflammation-associated																					
Inflammasome	Nlrp3	3.5	NS	3.9	1.54E-02	5.7	1.23E-03	6.7	9.08E-05	1.1	NS	1.6	NS	1.9	NS	1.4	NS	1.2	NS	1.7	NS
Il1 Superfamily	Il1b	3.5	NS	3.2	NS	3.9	3.91E-02	7.7	3.86E-04	−1.1	NS	1.1	NS	2.2	NS	1.2	NS	2.0	NS	2.4	NS
	Il33	1.8	NS	3.7	5.24E-03	2.5	NS	8.8	9.19E-08	2.1	NS	1.4	NS	4.9	6.46E-04	−1.5	NS	3.6	6.36E-03	2.4	NS
	Il36a	5.2	NS	7.4	NS	1.4	NS	12.1	3.56E-02	1.4	NS	−3.9	NS	2.3	NS	−5.5	NS	8.9	NS	1.6	NS
	Il36b	−2.3	NS	−4.8	1.09E-02	−2.8	NS	−4.2	8.21E-03	−2.1	NS	−1.2	NS	−1.8	NS	1.8	NS	−1.5	NS	1.1	NS
	Il36g	20.5	1.56E-02	19.5	6.95E-03	39.2	4.51E-04	78.4	5.76E-06	−1.0	NS	1.9	NS	3.8	NS	2.0	NS	2.0	NS	4.0	NS
Inflammation biomarkers	Lcn2	1.8	NS	2.1	NS	2.3	NS	7.5	1.01E-07	1.1	NS	1.3	NS	4.2	9.56E-04	1.1	NS	3.3	6.21E-03	3.6	1.90E-03
	S100a9	9.7	NS	7.7	NS	9.3	4.88E-02	34.6	3.21E-04	−1.3	NS	−1.0	NS	3.5	NS	1.2	NS	3.7	NS	4.5	NS
	S100a8	9.2	NS	5.4	NS	5.7	NS	21.1	2.96E-03	−1.7	NS	−1.6	NS	2.3	NS	1.1	NS	3.7	NS	3.9	NS
	Tnf	3.9	3.82E-03	6.7	1.64E-06	13.2	1.83E-11	26.5	1.54E-20	1.7	NS	3.3	3.47E-02	6.7	1.19E-06	2.0	NS	2.0	NS	4.0	3.49E-04

**Table 6:** Selected differentially expressed genes in the distal colon.

	**Statistical** ** code**	**A**		**B**		**C**		**D**		**K**		**L**		**M**		**Q**		**R**		**S**	
		**0I_0U**		**2I_0U**		**5I_0U**		**10I_0U**		**2I_0I**		**5I_0I**		**10I_0I**		**5I_2I**		**10I_5I**		**10I_2I**	
**Categorization**	**Gene**	**FC**	***p* (adj)**	**FC**	***p* (adj)**	**FC**	***p* (adj)**	**FC**	***p* (adj)**	**FC**	***p* (adj)**	**FC**	***p* (adj)**	**FC**	***p* (adj)**	**FC**	***p* (adj)**	**FC**	***p* (adj)**	**FC**	***p* (adj)**
T Helper cell type	Ifng	12.3	7.29E-04	56.1	4.08E-10	49.1	1.82E-09	37.8	5.56E-09	4.6	3.31E-02	4.0	NS	3.1	NS	−1.1	NS	−1.3	NS	−1.5	NS
Th1-associated	Il12a	−2.7	NS	−2.0	NS	−3.0	3.52E-02	−8.8	2.46E-05	1.4	NS	−1.1	NS	−3.3	NS	−1.6	NS	−2.9	NS	−4.5	7.08E-02
	Irf1	1.4	9.04E-04	2.3	7.20E-22	2.1	7.20E-17	2.3	3.68E-24	1.4	4.37E-03	1.3	NS	1.4	NS	−1.1	NS	1.1	NS	1.0	NS
	Stat1	2.4	1.51E-04	5.0	3.42E-16	4.3	2.04E-13	5.6	2.33E-20	2.1	7.02E-03	1.8	NS	2.4	3.13E-04	−1.2	NS	1.3	NS	1.1	NS
	Stat4	1.5	NS	2.7	1.68E-03	1.9	NS	1.0	NS	1.8	NS	1.2	NS	−1.5	NS	−1.4	NS	−1.8	NS	−2.6	2.60E-02
	Irf8	1.6	2.24E-03	2.4	4.66E-13	2.4	3.33E-12	2.8	2.82E-18	1.6	8.68E-03	1.5	4.83E-02	1.8	1.22E-04	−1.0	NS	1.2	NS	1.1	NS
	Ido1	12.5	1.86E-05	67.5	2.46E-16	63.1	9.71E-16	96.6	6.89E-21	5.4	1.78E-02	5.1	NS	7.7	9.96E-04	−1.1	NS	1.5	NS	1.4	NS
	Nos2	60.9	6.14E-35	175.2	2.17E-63	113.2	5.89E-53	108.0	1.22E-56	2.9	1.50E-02	1.9	NS	1.8	NS	−1.5	NS	−1.0	NS	−1.6	NS
TH17-assocuiated	Il17a	25.2	5.58E-03	119.1	1.52E-06	62.4	4.13E-05	36.7	2.35E-04	4.7	NS	2.5	NS	1.5	NS	−1.9	NS	−1.7	NS	−3.2	NS
	Il17c	4.7	2.84E-01	18.1	1.13E-02	11.6	3.45E-02	14.1	1.54E-02	3.8	NS	2.5	NS	3.0	NS	−1.6	NS	1.2	NS	−1.3	NS
	Il22	25.5	1.22E-02	98.9	2.96E-05	86.5	5.32E-05	39.9	5.88E-04	3.9	NS	3.4	NS	1.6	NS	−1.1	NS	−2.2	NS	−2.5	NS
	Il22ra2	−3.5	5.83E-02	−5.1	3.45E-03	−5.1	3.20E-03	−14.8	1.44E-07	−1.5	NS	−1.5	NS	−4.3	2.61E-02	−1.0	NS	−2.9	NS	−2.9	NS
Inflammation-associated																					
Inflammasome	Nlrp3	3.0	1.24E-03	7.8	1.35E-12	5.2	2.14E-08	4.9	1.06E-08	2.6	1.41E-02	1.7	NS	1.6	NS	−1.5	NS	−1.1	NS	−1.6	NS
Il1 Superfamily	Il1b	2.5	NS	16.9	2.44E-12	7.9	4.60E-07	7.8	1.04E-07	3.1	2.01E-02	3.1	NS	3.1	2.01E-02	−2.1	NS	−1.0	NS	−2.2	NS
	Il33	−1.0	NS	3.2	5.08E-04	1.7	1.30E-01	2.2	1.34E-02	2.3	3.32E-02	1.8	NS	2.3	3.32E-02	−1.9	NS	1.3	NS	−1.4	NS
	Il36a	18.7	1.76E-02	83.0	1.91E-05	45.4	2.79E-04	27.6	1.14E-03	4.4	NS	2.4	NS	1.5	NS	−1.8	NS	−1.6	NS	−3.0	NS
	Il36b	21.5	3.11E-02	16.5	2.85E-02	28.2	6.85E-03	32.3	2.92E-03	−1.3	NS	1.3	NS	1.5	NS	1.7	NS	1.1	NS	2.0	NS
	Il36g	19.5	2.83E-02	232.8	1.10E-06	53.6	5.17E-04	128.9	6.68E-06	11.9	5.21E-02	2.7	NS	6.6	NS	−4.3	NS	2.4	NS	−1.8	NS
Inflammation biomarkers	Lcn2	2.7	NS	10.6	1.41E-07	5.7	1.28E-04	14.6	2.21E-10	3.9	2.73E-02	2.1	NS	5.3	1.74E-03	−1.8	NS	2.5	NS	1.4	NS
	S100a9	94.5	6.85E-05	979.6	8.83E-12	325.3	1.38E-08	609.9	3.92E-11	10.4	4.58E-02	3.4	NS	6.5	NS	−3.0	NS	1.9	NS	−1.6	NS
	S100a8	180.2	1.20E-06	1105.2	3.05E-13	346.9	1.66E-09	445.8	4.55E-11	6.1	NS	1.9	NS	2.5	NS	−3.2	NS	1.3	NS	−2.5	NS
	Tnf	10.7	7.05E-21	17.4	2.35E-27	16.9	1.03E-26	13.8	2.33E-25	2.2	2.82E-02	2.1	NS	1.7	NS	−1.0	NS	−1.2	NS	−1.3	NS

**Table 7:** Differentially expressed T-cell associated genes in the distal colon.

**Statistical code**	**A**		**B**		**C**		**D**		**K**		**L**		**M**		**Q**		**R**		**S**	
	**0I_0U**		**2I_0U**		**5I_0U**		**10I_0U**		**2I_0I**		**5I_0I**		**10I_0I**		**5I_2I**		**10I_5I**		**10I_2I**	
**Gene**	**FC**	***p* (adj)**	**FC**	***p* (adj)**	**FC**	***p* (adj)**	**FC**	***p* (adj)**	**FC**	***p* (adj)**	**FC**	***p* (adj)**	**FC**	***p* (adj)**	**FC**	***p* (adj)**	**FC**	***p* (adj)**	**FC**	***p* (adj)**
Cd3d	2.6	4.56E-03	4.1	8.70E-07	3.4	1.89E-05	1.8	NS	1.6	NS	1.3	NS	−1.5	NS	−1.2	NS	−2.0	NS	−2.3	3.71E-02
Cd3e	2.5	1.34E-03	3.6	3.04E-07	3.1	9.59E-06	1.4	NS	1.4	NS	1.2	NS	−1.9	3.67E-02	−1.2	NS	−1.1	NS	−2.7	2.53E-03
Cd3g	3.2	1.15E-04	4.2	7.29E-08	3.6	1.97E-06	1.7	4.63E-02	1.3	NS	1.1	NS	−1.9	4.66E-02	−1.2	NS	−2.1	NS	−2.5	1.36E-02
Cd4	1.5	NS	1.9	1.46E-02	1.5	NS	−1.4	NS	1.3	NS	1.0	NS	−2.0	1.79E-02	−1.3	NS	1.1	NS	−2.6	3.16E-03
CD8a	4.9	4.81E-05	5.9	2.48E-07	5.6	6.89E-07	2.2	2.34E-02	1.2	NS	1.1	NS	−2.2	4.97E-02	−1.1	NS	−1.2	NS	−2.7	4.35E-02
CD28	1.9	5.00E-02	2.8	1.73E-04	2.2	4.01E-03	1.2	NS	1.5	NS	1.2	NS	−1.7	NS	−1.3	NS	1.2	NS	−2.4	1.73E-02
Cd247	2.2	3.28E-02	3.0	2.40E-04	2.5	3.17E-03	1.3	NS	1.4	NS	1.1	NS	−1.7	NS	−1.2	NS	−1.9	NS	−2.3	4.87E-02
Ctla4	2.4	5.77E-03	4.9	1.27E-09	3.0	3.28E-05	1.5	NS	2.1	3.80E−02	1.3	NS	−1.6	NS	−1.6	NS	−2.0	NS	−3.3	1.60E-04
Gzma	30.6	9.68E-18	61.0	1.57E-28	40.1	5.94E-23	15.9	4.46E-14	2.0	NS	1.3	NS	−1.9	NS	−1.5	NS	2.2	NS	−3.8	9.52E-05
Gzmb	15.4	1.57E-13	50.2	1.79E-31	32.0	1.21E-24	18.2	7.68E-19	3.3	3.83E−03	2.1	NS	1.2	NS	−1.6	NS	1.5	NS	−2.8	1.13E-02
Il2ra	1.3	NS	2.8	5.93E-07	1.7	1.49E-02	1.2	NS	2.1	8.66E−03	1.3	NS	−1.1	NS	−1.6	NS	−1.4	NS	−2.2	2.28E-03
Il2rb	1.5	NS	2.8	3.16E-07	2.0	4.83E-04	1.2	NS	1.8	3.19E−02	1.3	NS	−1.3	NS	−1.4	NS	−1.8	NS	−2.4	4.34E-04
Il2rg	1.2	NS	2.4	1.06E-02	1.2	NS	−1.4	NS	2.0	NS	1.0	NS	−1.7	NS	−2.0	NS	−1.7	NS	−3.3	8.60E-03
Lat	3.3	7.52E-05	5.4	1.36E-10	3.8	6.37E-07	2.4	6.32E-04	1.6	NS	1.1	NS	−1.4	NS	−1.4	NS	−1.6	NS	−2.2	2.89E-02
Lck	1.7	1.20E-02	2.2	7.32E-06	1.9	3.83E-04	1.2	NS	1.3	NS	1.1	NS	−1.4	NS	−1.2	NS	−1.6	NS	−1.9	7.80E-03
Trac	2.8	1.95E-03	3.9	1.78E-06	3.4	2.44E-05	1.6	NS	1.4	NS	1.2	NS	−1.8	NS	−1.2	NS	−2.2	NS	−2.5	2.01E-02
Trbc1	2.0	2.57E-02	2.1	4.81E-03	2.1	6.62E-03	−1.2	NS	1.1	NS	1.0	NS	−2.4	3.85E-03	−1.0	NS	−2.5	2.37E-02	−2.6	6.50E-03
Trbc2	1.9	2.37E-02	3.2	2.04E-06	2.1	2.80E-03	1.5	NS	1.6	NS	1.1	NS	−1.3	NS	−1.5	NS	−1.5	NS	−2.2	1.91E-02
Trdc	−1.6	NS	−1.1	NS	−2.4	2.68E-02	−3.0	2.66E-03	1.4	NS	−1.5	NS	−1.9	NS	−2.2	NS	−1.2	NS	−2.7	NS
Trgc1	−1.1	NS	1.5	NS	−1.3	NS	−1.8	NS	1.7	NS	−1.1	NS	−1.6	NS	−1.8	NS	−1.4	NS	−2.6	NS
Trgc2	1.1	NS	1.2	NS	1.7	NS	−1.7	NS	1.0	NS	1.5	NS	−2.0	NS	1.4	NS	−3.0	NS	−2.1	NS
Trgc3	1.9	NS	2.4	NS	1.0	NS	1.0	NS	1.3	NS	−1.7	NS	−2.0	NS	−2.1	NS	1.0	NS	−2.6	NS
Trgc4	−2.4	9.81E-03	−1.9	3.26E-02	−2.4	3.63E-03	−5.8	2.62E-09	1.3	NS	1.0	NS	−2.4	2.88E-02	−1.3	NS	−2.4	NS	−3.1	1.20E-02
Zap70	2.0	1.32E-02	2.8	3.25E-05	2.5	2.44E-04	1.1	NS	1.4	NS	1.2	NS	−1.9	2.95E-02	−1.1	NS	1.3	NS	−2.6	3.10E-03

## Discussion

The results presented here confirm and extend on our previous results (7) that mice fed a diet containing RPS that emulates a typical American diet for both macro and micronutrients (22) can have significant effects on the production of butyrate as well as the microbiota and gene expression in the cecum and colon in a dose-dependent manner. The previous results suggested that consumption of resistant starch, possible by raising levels of SCFAs including butyrate, may prime the immune response to bacteria, helminth parasites and virus. Thus, we anticipated that feeding RPS diets, would improve the host response against a bacterial challenge by *Cr* that targets the cecum and colon, and whose growth has been shown to be inhibited by high concentrations of butyrate (34). After colonizing the cecum, *Cr* colonizes the DC around day 3 and 4 post-infection. Interestingly, we found that feeding RPS diets improved the ability of *Cr* to colonize the DC early in infection by increasing both the number of mice productively infected and the *Cr* burden at day 4 post-infection. Increased variability in *Cr* burden early in the infection cycle (days 3 and 4 post-infection) has been reported in mice fed a low-fiber purified high-fat diet but not in higher fiber chow-fed mice (53). A study using a type 2 resistant corn starch, however, did not observe increased fecal excretion of *Cr* early in infection but used an AIN-93G basal diet rather than a high-fat diet (38). This also raises the possibility that different starches, even within the same category could act differently.

We also found higher levels of colon tissue associated *Cr* on day 12 post-infection only in mice fed the 10% RPS diet that was associated with increased pathology. Furthermore, infected mice fed the 10% RPS diet had enlarged spleens 12 days post-infection that is indicative of a systemic response to infection. Our results are in agreement with An et al. (28) where they also saw increased numbers of productively infected mice early in infection in mice fed a standard high-fiber grain-based rodent chow or a Western-style high fat diet supplemented with inulin compared to the low-fiber Western-style diet, as well as a trend toward increased crypt length and spleen size in chow or inulin supplemented diet (28). This strongly suggests that diets containing fermentable substrates enhance the ability of *Cr* to colonize the host.

Two interconnected variables could lead to the increased *Cr* colonization. *EHEC, EPEC*, and *Cr* virulence genes are encoded in several Locus of Enterocyte Effacement (LEE) operons that are responsible for many of the pathogenic effects of these organisms (54). As we and others have shown, consumption of RS results in increased levels of butyrate (4, 7, 17, 55). Low levels of short-chain fatty acids, especially butyrate, can enhance the growth and virulence of *EHEC* (56) while others have shown that high levels of butyrate can inhibit growth of *Cr in vitro* and pathology *in vivo* (34, 39). Feeding RS to mice produces SCFAs which activate intestinal gluconeogenesis leading to increased production of succinate (1) which can activate the expression of the LEE. Thus, it is possible that butyrate can produce divergent effects depending on the luminal concentration with low levels possible enhancing colonization and pathology while high levels are inhibitory.

Several other products resulting from breakdown and fermentation of fibers and RS can impact EHEC, EPEC, and *Cr*. Mono- and polysaccharides are generated from dietary fibers and RS by a host of bacterial enzymes (57) that can impact the growth of EHEC, EPEC, and *Cr*. Both *Cr* and *E. coli* grew best on monosaccharides while the common commensal bacteria *B. thetaiotaomicron* and *B. vulgatus* could catabolize both mono-and polysaccharides (58). Thus, breakdown of fibers and RS may provide *Cr* and pathogenic *E. coli* an increased source of monosaccharides for catabolism which is important for their early growth required for establishing an infection before the switch to gluconeogenesis required for maintaining colonization (59). Additional metabolomic studies will need to be undertaken to further explore the colonic environment resulting from feeding RPS and how it relates to the increased RPS-induced *Cr* colonization.

Uninfected mice had a dose-dependent increase in DC crypt length in response to feeding dietary RPS (Figure 1B) and has been observed in pigs fed RPS (20). *Cr* is known to induce colonic hyperplasia (51) that was also observed in our study (Figure 1B) and an LC-MS/MS analysis of protein extracts of *Cr*-infected intestinal epithelial cells showed upregulation of proteins associated with the cell cycle, ribosome biogenesis and DNA replication (53). In agreement with these findings, more cell cycle associated genes were induced in the DC by infection in RPS-treated mice compared to infected mice fed the basal diet (Supplementary Tables 10, 11).

We previously showed that dietary RPS induced dose-dependent changes in the cecal microbiota that were most prevalent in mice fed the 10% RPS diet (7). The α-diversity plots of cecal and fecal contents from uninfected mice showed similar trends with decreasing diversity associated with increasing dietary RPS that has been reported in rodents (4, 60–63) and pigs (64). Infection, however, affected the cecal and fecal α-diversity differently with the cecal contents showing both a significant treatment (diet) and infection effect. In contrast, fecal α-diversity in infected mice was only significantly affected by diet and not by infection. The latter result was surprising in that we expected more of an effect of infection on day 6 fecal samples when the *Cr* burden is at or near peak compared to the cecum where there is no significant *Cr* burden at day 12 post-infection.

The α-diversity results (Figure 3) can be partially explained by the fact that in the cecal contents the *Lachnospiraceae NKA136 group* dominates the microbiota achieving a relative abundance of approximately 60% in uninfected mice fed the 10% RPS diet, indicating a growth advantage for this genus in the presence of high levels of RPS that drives down diversity (Supplementary Figure 5A; Table 1). This dominance is reduced by approximately 50% by infection allowing other genera to increase, thus increasing diversity and contributing to the significant effect of infection on α-diversity in the cecal contents. Similar results for the *Lachnospiraceae NKA136 group* were observed in the feces obtained at 6 days post-infection when the *Cr* burden is at or near peak levels. However, compared to the cecal contents *Lachnospiraceae NKA136 group* relative abundance only reached 35% in the feces of uninfected mice (Supplementary Figure 5B, Table 2). In addition, a second genus, *Faecalbaculum* was present at a much higher relative abundance in feces of RPS fed mice, reaching a comparable relative abundance of approximately 30% in uninfected mice fed the 10% RPS diet. Thus, these two genera dominate the fecal microbiota of RPS fed mice. As observed in the cecal contents, infection reduced the fecal content of *Lachnospiraceae NKA136 group* by about 50%, again indicating that infection mitigated some of the growth advantage awarded *Lachnospiraceae NKA136 group* by feeding RPS increasing the opportunity for other genera to fill the void but the effect in the feces is significantly smaller. In addition, the other dominate genus, *Faecalbaculum*, relative abundance was not reduced by infection. This likely contributed to the reduced effect of infection on the fecal microbiota as well. Nevertheless, the fact that infection had such an impact on the cecal microbiota, even after the cessation of active *Cr* growth in the cecum, strongly suggests that *Cr* infection has long lasting effects on the cecal microbiota.

Although the α-diversity in uninfected and infected D6 fecal samples was similar and dominated by dietary effects (Figure 3), the relative abundance of only a few genera were different between infected and uninfected mice including the aforementioned *Lachnospiraceae NKA136 group* and *Erwinia*, which was only found in infected mice (Table 2). The selective growth of Erwinia in infected animals may be related to its ability to grow in the aerobic environment caused by a *Cr* infection induced change in epithelial cell metabolism shifting metabolism form oxidative phosphorylation to glycolysis which increased the availability of oxygen which favors the growth of *Cr* and other *Enterobacteriaceae* (65). PCA plots (Figure 4), nevertheless, showed separation of the samples by dietary RPS levels and infection with different treatment/infection groups clustering together showing an effect of both diet and infection on the groupings. PCA plots of cecal samples (Figure 4C) had separation by infection and diet although the separation due to infection was less pronounced than in feces. However, while the overall p value for a PERMANOVA analysis of the fecal and cecal data was highly significant (*p* < 0.001) after Bonferroni correction individual comparisons did not show any significant changes in fecal samples (data not shown).

LEfSe analysis (Figure 6) identified shared discriminating genera between cecal contents and feces. *Lachnospiraceae NK4A136 group* was discriminating for uninfected mice fed 10% RPS in both cecal contents and feces while *Faecalibaculum* and *Bacteroides* were discriminating for 10% RPS fed infected mice in both cecal contents and feces. There was a surprising lack of discriminating genera in feces for uninfected and infected mice fed the 5% RPS diet. The reasons for this are not clear. Nor were there any shared discriminating genera between cecal contents and feces for uninfected and infected mice fed the 2% RPS diets. Cecal contents and feces from uninfected mice fed the basal, 0% RPS diet shared several discriminating bacteria including *Clostridium senso stricto 1, Blautia, Bilophia*, and *Turcibacter* but infected mice did not share discriminating genera between the cecal contents and feces. However, many discriminating genera from uninfected and infected cecal contents and feces from mice fed the 0% RPS diet did share the characteristics of having decreased relative abundance as the RPS dose increased. In contrast, the top discriminating genera in cecal contents from uninfected and infected mice fed the 5 and 10% had increased relative abundance compared to mice fed the 0% RPS diet. Similarly, the top discriminating genera in feces obtained from uninfected and infected mice fed 2 or 10% RPS also had increased relative abundance compared to mice fed the 0% RPS diet.

The relative similarity of the fecal microbiota between uninfected and *Cr*-infected mice has been reported by others (53). Interestingly, changes were found in the composition of mucosa-associated microbiota between uninfected and *Cr*-infected mice suggesting that these changes may be of greater importance (53). Other studies have shown differences in mucosa vs. luminal microbiota composition (66) as well as differences in the mucosa-associated microbiota at different locations with the large intestine (67). Our results suggest that the luminal microbiota composition in the DC (Table 2) may be dominated by what occurs in the cecum (Table 1). In mice, the primary site of fermentation of resistant starches is the cecum (68) and consumption of RPS had a significant impact on the cecal microbiota [(7) and this study] and many of the diet induced changes in the cecal microbiota are also observed in the fecal microbiota. This indicates that diet induced changes to the cecal microbiota are having a major effect on the composition of the fecal microbiota, and that fecal samples may provide a good approximation of diet-induced changes in the cecum.

We also looked at gene expression in the cecum and DC of mice fed the basal TWD as well as the TWD supplemented with RPS to identify potential mechanisms associated with increased colon hyperplasia and colonization. In the DC and to a lesser extent the cecum, Reactome Pathway analysis (Supplementary Tables 8, 10) and our own assessment from the PIN database revealed that the combination of RPS and infection led to the induction of a very large number of genes involved in the cell cycle (Supplementary Tables 9, 11) with a greater number of genes induced in the DC vs. the cecum. The increase in cell cycle genes is particularly remarkable given the overall decrease in gene expression by 10% RPS the DC and colon and reflects the infection induced increase in colon/bodyweight ratio and hyperplasia observed in the distal colon (Figures 1A, B). The lower effect in cecum may be due to lower levels of *Cr* by D12 post-infection not driving the hyperplastic response to infection. In the DC, RPS alone led to a modest increase in induction of some of these genes in the 2, 5, and 10% group respectively (3, 26, 69) that correlated with increased crypt length in uninfected mice (Figure 1B). Although the average level of induction of these changes ranged from 2.1 to 2.3-fold, the sheer number of them undoubtedly leads to synergy. Breakdown of the specific cell cycle phase revealed that G1/S Transition Phase and S Phase were most affected by RPS with the enrichment scores increasing by 1.5–1.7 with the addition of RPS. The M phase was most affected by infection and RPS, inducing approximately 2/3 of the genes in the cell cycle.

This effect was not homogeneous as several different patterns were observed for genes involved in the cell cycle. Except for the 5I_0Uinf comparison, the average level of induction in the infected group increased with increased RPS concentrations in the diet with the average being 1.54, 2.13, 2.03 and 2.30-fold in the 0, 2, 5% and 10% RPS groups, respectively and as similar pattern in crypt length was observed in infected mice. The same biphasic pattern was evident for the average significance level of genes (1.2E-02, 7.2E-04, 1.3E-03, 1.9E-04), and the number of significant genes expressed at a ≥ 1.5-fold level (146, 296, 282 and 332). Several commonly used markers of cell activation and proliferation, the antigen identified by the mAb antibody Ki-67 (Mki67) (70) and proliferating cell nuclear antigen (Pcna) also exhibited a biphasic expression pattern in response to RPS; however, some genes, did not obey this pattern including the transferrin receptor (Tfrc) (71). It was upregulated in the DC of all RPS-treated mice vs. control. It was also increased by infection but was not increased by infection in RPS-treated animals. It is not known whether these changes in gene expression reflect division of parenchymal cells *in situ* or infiltration of dividing cells but the increase in crypt depth described above suggest the former mechanism may be operative.

Several cytokines are important for controlling *Cr* infections. The type 2 interferon, interferon-g (Ifng) is the principle driver of Th1 responses (72) and has been shown to be important for clearance of *Cr* (73, 74). It is produced by a variety of cell types including T cells. Ifng was significantly upregulated [57.7 (*p* = 5.46E-04), 83.9 (*p* = 1.71E-05), 160.3 (*p* = 3.90E-07) and 276.8-fold (*p* = 1.96E-09)] in a dose-dependent fashion in the 0 I_0U, 2I_0U, 5I_0U and 10I_0Ucomparisons in the cecum (Table 6).

Two type 2 IFN-induced genes, indoleamine 2,3-dioxygenase 1 (Ido1) and Ido2 (69) also exhibited dose response patterns increased from 3.5, 5.4, 10.4 and 20.2 in the 0I_0U, 2I_0U, 5I_0U, and 10I_0U comparisons, respectively for Ido1 (Table 6). Ido1 and Ido2 metabolize L- tryptophan into kynurenine, which can suppress inflammation and immune responses including those to *Cr* (75).

In DC, Ifng was significantly induced by infection (12.3-fold, *p* = 7.29E-04). 2% RPS increased infection-induced expression [4.6-fold (to 56.1-fold, *p* = 4.08E-10)] but 5 and 10% were progressively less effective increasing it only increasing it [4.0-fold (to 49.1 fold, p = 1.82E-09)], and [3.0-fold (to 37.8-fold, p = 5.56E-09)] (Table 6). In contrast to this, Ido1 exhibited a pattern where the highest expression was seen in the 10% RPS group (96.6-fold) followed by 2% (67.5-fold), 5% (63.1-fold) and 0%. (12.5-fold). It is tempting to speculate that overproduction of Ido1 in the cecum and DC contributes to the enhanced bacterial colonization seen in response to increasing levels of RPS in our model. Interleukin 12a, like Ifng is critical for mediating immune response to Cr (73). Il12a expression did not change due to Inf or RPS status in cecum (Table 5); however, in DC, IL12a was down regulated in the 5I_0U (-3.0 fold) and 10I_0U (-8.8 fold) comparisons (Table 6).

IL-17a is produced by type 3 innate lymphoid (ILC3) cells, neutrophils and TH17 cells, is the principle driver of Th17-associated responses (76), and is important for the control of *Cr* infection (77). A related cytokine, IL-17c is produced by dendritic cells, macrophages and T cells and is associated with inflammatory responses (78). The IL-17c receptor is essential for protection against *Cr* colonization and mortality (79). A pattern of expression similar to Ifng was observed for IL17a and Il17c in the DC (Table 6) but not the cecum (Table 7). In the cecum, Il17a was non-significantly upregulated in all four RPS treatment groups vs. the 0U group. In the cecum, Il17c was non-significantly upregulated in three treatment groups vs. the 0U group. It only reached statistical significance [6.3-fold (*p* = 3.77E-02)] in the 10I_0U comparison. In DC, Il17a mRNA was significantly induced by infection (25.2-fold, *p* = 5.58E-03). 2% RPS increased infection-induced expression [4.7-fold (to 119.1-fold, *p* = 1.52E-06)] but 5% and 10% were progressively less effective at increasing it; [1.9-fold (to 62.4-fold, *p* = 4.13E-05)] and [3.2-fold (to 36.7-fold, *p* = 2.35E-04)], respectively. A lower IL-17A response in DC may have contributed to the increased colonization observed at 12-days post-infection (Figure 2B).

Similarly, but to a lesser extent, Il17c was significantly induced by infection (4.7-fold, *p* = 2.84E-01). 2% RPS increased infection-induced expression [3.8-fold (to 18.1-fold, p = 1.13E-02)] but 5% and 10% were progressively less effective increasing it only increasing it [2.5 fold (to 11.6-fold, *p* = 3.45E-02)] and [3 fold (to 14.1-fold, *p* = 1.54E-02)], respectively. As expected, infection induces a Th17 response and this response is enhanced by RPS, but the effect was not dose-dependent given that the 2% diet induced the greatest potentiation.

Interleukin-22 is produced by ILC3 cells, NK cells and TH17 cells (80, 81) and is important for controlling *Cr* infections (27). It is a positive regulator of inflammation and is associated with Th1(82) and Th17-responses (83). Il22 was upregulated (25.5 (NS), 44.0, 36.6, 61.1) and one of its receptors, Il22ra2, downregulated [– (1.8 (NS), −3.4, −3.1 −33.6)] in a biphasic fashion in the 0 I_0, 2I_0, 5I_0 or 10I_0 groups compared to 0 I_0. Similarly in DC, Il22 was upregulated (25.5 (p = 1.22E-02), 98.9 (2.96E-05), 86.5 (p = 5.32E-05), 61.1 (*p* = 5.88E-04) and Il22ra2, downregulated (−3.5 (*p* = 5.83E−02), −5.1 (p = 3.45E−03), −5.1 (*p* = 3.20E−03) −14.8 (1.44E−07) fold in the 0 I_0, 2I_0, 5I_0 or 10I_0 groups compared to 0 I_0. Il22ra2 is a decoy receptor and acts as an Il22 receptor antagonist (84) the expected biological response would be to increase the local production and activity of IL-22 in both tissues.

Multiple markers of inflammation were also found to be differentially expressed due to diet and infection in cecum (Table 5) and DC (Table 6). Interleukin 1b (Il1b) and Tnf were significantly higher in the 5I_0I, 10I_0I, and 10I_2I comparisons, indicating a greater level of inflammation in the infected animals fed 10% RPS. Fecal lipocalin 2 (Lcn2/NGAL) is used as a marker of intestinal inflammation (85). Similarly, neutrophil-derived calprotectin (S100a9) and calprotectin L (S100a8) are used as fecal markers of inflammation (86). In cecum, Lcn2 (7.5-fold) and S100a8 (21.2-fold) were only significantly upregulated in the 10I_0U comparison. S100a9 (34.6-fold) were only significantly upregulated in the 5nf_0U (9.3 fold) and 10I_0U (34.6 fold) comparisons. Thus, it appears that higher dose of RPS exacerbates inflammation in the cecum of *Cr* infected mice. In contrast in DC, Lcn2, S100a8 and S100a9 exhibit a biphasic pattern of response. Il1b and Tnf expression are higher in the 2I_0U comparison followed by 5I_0U, 10I_0U and 0I_0U.

In the DC, DAVID Analysis of Reactome pathways revealed a rough doubling of the number of genes involved in “Immune System” “Neutrophil Degranulation” and “Adaptive Immune System”, pathways by RPS, regardless of the dose (Supplementary Table 6). The enrichment scores for these pathways were relative similar to the 0% RPS group. In infected animals, the number of genes in the “Cytokine Signaling in Immune System” pathway was tripled and the enrichment score was increased by 50%, in the 2% RPS vs. 0% Inf comparison; however, the number of genes, enrichment scores and statistical significance were all lower in the 5% and 10% groups vs. the 2% group.

Resident T cells in the GALT of the colon play an important role in the response to pathogens (87). Analysis of DEGs in DC, revealed that infection (0I_0U) leads to a significant increase in 15/24 T cell-associated genes (Table 7). The increase appears to be mediated by a/b, CD8^+^ T cells. This could reflect *in situ* proliferation, infiltration of circulating T cells or impaired migration of T cells out of the tissue. The ratio is increased to 19/24 and 17/24 genes in the 2I_ 0U and 5I_0U groups, respectively; however, in the 10I_0U comparison, only 8/24 genes are significant. In the comparison of 10I_2I, 20/24 genes are downregulated. By pathway analysis, in DC, genes associated with TCR signaling were enriched 2.1-fold by infection, but this was not statistically significant. 2% RPS significantly increased it to 3.5-fold, but 5% and 10% only increased it to 3.3- and 2.7-fold, respectively. A progressive decrease in REACTOME enrichment scores (and statistical significance) were also observed for the following T Cell-related pathways; Translocation of ZAP-70 to Immunological Synapse, CD28 Family Co-Stimulation and Transcriptional Regulation by RUNX1 (Supplementary Table 16).

Based upon gene expression profiles, we found significant changes associated with B cells. Resident B cells in the follicle-associated epithelium of the cecum and colon are associated with the gut-associated lymphoid tissue (GALT) and play an important role in the response to pathogens (88). Immunoglobulin-associated genes constitute 10 out of the top 20 upregulated genes in the cecum 0I_0U comparison (Table 3); however, they are missing from the DC top 20 comparison (Table 4). Analysis of DEGs in DC, revealed that infection (0I_0U) leads to a significant, low-level increase in 3/16 generic B cell-associated genes. Two out of 16 genes are downregulated. The increase appears to be mediated by IgA producing B cells. The ratio of upregulated genes is increased to 5/16 and 3/16 genes in the 2I_ 0U and 5I_0U groups, respectively. The ratio of downregulated genes to 0/16 and 2/16 genes in the 2I_ 0U and 5I_0U groups, respectively. In the 10I_0U comparison, 0/16 genes are significantly upregulated and 7/16 are downregulated. Ighd and Ighm mRNA were exclusively downregulated in the 10% Inf DC. In the comparison of 10I_2I, 8/16 genes were downregulated.

Others have reported metabolic changes induced by consumption of RS and by *Cr* infection that were predicative of increased colonization (89). In our studies, in DC DAVID Analysis of Reactome pathways revealed a doubling of the number of genes involved in “Metabolism”, “Carbohydrate Metabolism”, “Glucose Metabolism”, “Glycolysis”, “Nucleotide Metabolism” pathways by RPS, regardless of the dose; however only the latter 3 pathways were statistically significant (Supplementary Table 6). Intestinal expression of several of these genes that were increased in a biphasic manner by RPS in our experiment (Slc5a9, Ldha, Slc16a3) also correlated with the transition from oxidative phosphorylation to aerobic glycolysis, and severe disease in animals infected with *Cr* (90).

In addition to these and Ido1-mediated tryptophan degradation, we identified several additional metabolic pathways, that were differentially regulated by RPS. The “Biosynthesis of docosahexaenoic acid (DHA)-derived specialized pro-resolving mediators (SPMs)” pathway was selectively (*p* = 1.02E−03) downregulated in the 10I_0U comparison (Supplementary Table 6). The 12 genes in that pathway Gstm4 (−1.9), Cyp2d22 (−7.8), Cyp2c66 (−2.0), Hpgd (−4.7), Gpx4 (−1.5), Cyp2c65 (−2.9), Alox5 (−2.7), Ephx2 (−4.7), Cyp1a1 (−7.9), Alox12 (−1.6), Cyp2e1 (−4.6), Ltc4s (−2.3) were modestly downregulated in the 10I _0U comparison. Four of these genes, Cyp2d22 (−2.9), Hpgd (−2.6) Ephx2 (−2.1) and Alox12 (−2.0), were also downregulated in the 5I_0U comparison. Two of these genes were downregulated in the 2I_0U [Cyp2d22 (−2.5) Ephx2 (−2.0)] and the 0I_0U [Cyp2d22 (2.0) Ephx2 (−1.6)] comparisons, respectively. Specialized pro-resolving mediators, such as DHA-derived lipoxins and maresins, are potent inhibitors of the inflammatory response but also serves to increase antibacterial responses *via* increased macrophage and neutrophil phagocytosis (91). Importantly, the SPMs, resolvin D1 and resolvin D5 reduced bacterial loads, mitigated neutrophil infiltration and were protective against death in mice infected with *Cr* (89).

A change in several other genes with mechanistic potential occurred after feeding RPS to *Cr*-infected animals. Phenazine biosynthesis-like protein domain containing 1 (Pbld1), a negative regulator of NF-KB activation (92) was one of the top 20, 10%-downregulated genes in cecum; its expression decreased significantly, in a dose-dependent fashion only in the *Cr*-infected, RPS-fed animals; 2% (−9.7–fold), 5% (−14.4–fold) and 10% (−69.0–fold). It's expression in DC, fit the biphasic pattern as described above for other genes; 0% (−5.8–fold), 2% (−17.9–fold), 5% (−8.8–fold) and 10% (−19.4–fold). Pbld1 expression is decreased in mice and humans with colitis (92)and Pbld1-knockout mice are more susceptible to experimental colitis-induced inflammation. Leucine rich repeat and Ig domain containing 2 (Lingo2), a Trefoil factor 3 (TFF3) receptor (93), was highly downregulated in the cecum of 5% (−4.8–fold) and 10% (−37.6–fold) RPS groups. In DC, it was only downregulated (−3.7–fold) in the 10% group. Lingo2 knockout mice are highly susceptible to experimental colitis (93).

The 16 genes that were downregulated by RPS in DC are involved in all stages of VA metabolism, from cellular uptake to proteins involved in the biological activity of retinoic acid (RA), it's most biologically active metabolite. The lone receptor for the retinol delivery protein, Stra6 (94) (not shown in Supplementary Figure 8) was down regulated in the 10% RPS group. The mRNA for beta-carotene oxygenase 2 (Bco2) (95), one of two enzymes that convert beta carotene into retinol, were downregulated by all 3 doses of RPS (2%, 5%, 10%). The genes for the enterocyte-specific, intracellular retinol transport protein, Rbp2 (96), 5 retinol dehydrogenases, Adh1 (2%, 5%, 10%) (97) Rdh5 (2, 5, and 10%), Rdh7 (10%) Rdh12 (10%) and Dhrs3 (10%) (98), retinol saturase (Retsat) (not shown in Supplementary Figure 8), and the retinol esterification enzyme, (Lrat) (2, 5, 10%) (99) were down regulated by RPS. Three of the enzymes that convert retinal to retinoic acid (RA), Cyp1a1(100) (2, 5, 10%), Aox1 (101) (5, 10%), Aldh1a1 (10%), 1 of the 2 intracellular binding proteins for RA (28132904), Crabp1 (2, 5, 10%), and two out of 3 genes for RA receptors, Rarb (2, 5, 10%) Rarg (2, 5, 10%), were downregulated by RPS. There were changes in other mRNA for genes involved in VA metabolism, 2 retinol dehydrogenases, Rdh10 and Dhrs9 were upregulated by RPS while several other genes (Rdh1, Rdh9, Rdh13, Rdh14, Rdh16, Rdh19, Dhrs4, Dhrs7, Dhrs7c) were not affected. VA is essential for the differentiation of gut epithelial cells, T cells, B cells and macrophages and in the form of RA, is required for the IL-17-dependent immune response to *Cr* (102, 103), and may have contributed to the increase in colonization observed in RPS fed mice (Figures 2A, B).

## Conclusion

Our finding of increased colonization of *Cr* in animals fed 10% RPS, especially in the context of a largely intact Th1/Th17 response, is somewhat unexpected given our previous observations that animals fed RPS exhibited a dose-dependent increase in mRNA in genes associated with certain antibacterial responses (7). In addition, 5% RPS-fed pigs exhibited a reduction in Salmonella fecal shedding, different bacterial community compositions, and favorable cecal short chain fatty acid (SCFA) profiles relative to control animals (64). What is not clear from our study is what role, if any, the microbiota is contributing to the negative effect of consuming high levels of RPS on a subsequent *Cr* infection. Loss of microbiome diversity is thought to be deleterious to the host and mice fed the 10% RPS diet had the largest decrease in diversity accompanied the worst outcome in response to a *Cr* infection. *Cr* attachment to the mucosa is required for pathology and further research will be needed to determine if RPS consumption alters the composition of mucosa-associated commensal microbiota and how it impacts production of microbial metabolites that may be affecting *Cr* and cells composing the host mucosa.

*Via* RNASeq analysis, we have identified several host-mediated mechanistic pathways that could be associated with the increased colonization of *Cr* observed the animals fed 10% RPS. Specifically, in DC, we found a decrease in enrichment for genes associated with T cells, B cells, genes associated with the synthesis of DHA-derived SPMs and VA metabolism/retinoic acid signaling. We also found an increase in the expression of the potentially immunosuppressive gene, Ido1. There are several limitations to our approach. First, we measure gene expression and not actual biochemical or physiological functions. It is unknown whether the changes in genes associated with the 10% level are a cause of, or a result of, increased infection. Furthermore, we measured gene expression in whole tissue, where lymphoid and myeloid cells are infiltrating and not the principal cell type as would be found in draining lymph node. We also failed to characterize the various metabolic pathways identified by differential gene expression. These are the subject of our current investigations. Nevertheless, we provide compelling evidence that high level consumption of RPS, in the context of a typical American diet, may increase susceptibility to certain gastrointestinal bacterial infections.

## Data availability statement

The original contributions presented in the study are publicly available. This data can be found here: https://www.ncbi.nlm.nih.gov/bioproject/PRJNA757013.

## Ethics statement

The animal study was reviewed and approved by Beltsville Animal Care and Use Committee, USDA/ARS.

## Author contributions

AS and HD conceived and designed these experiments and supervised all experimental works. AS, CC, LC, and HD performed the experiments. AS, CC, and HD performed data analysis, drafted the manuscript, and helped revise the manuscript. All authors have read and approved the final manuscript.
