# Supplementary material for: Raw potato starch alters the microbiome, colon and cecal gene expression, and resistance to Citrobacter rodentium infection in mice fed a Western diet
Source: Front Nutr. 2023 Jan 10;9:1057318. doi: 10.3389/fnut.2022.1057318 (PMC9871501; doi:10.3389/fnut.2022.1057318)
Supplement: Supplementary file 14 [file Data_Sheet_1.DOCX]

Suppl. Figures

**Suppl. Figure 1. Body weights of uninfected and infected mice fed different levels of dietary RPS.** Mice were fed the TWD for 6 weeks and then switch to TWD diets containing 0, 2, 5, or 10% RPS for an additional 3 weeks, n=10/group A) and then infected with *Cr*, n=5/group B). Body weights were periodically taken and expressed as the percentage of the day 0 body weight when mice were either switched to the RPS containing diets or infected with *Cr*. Values are the mean and SEM, n=5/group.

**Suppl. Figure 2. Diet but not infection altered fecal pH.** Fecal pH was significantly decreased by consumption of a 10% RPS diet but was not further affected by *Cr* infection n=5-6/group. Data was analyzed by a two-way ANOVA. For each panel, bars are the mean ± SEM, groups with different letters are significantly different, p<0.05.

**Suppl. Figure 3. Mice fed a 10% RPS diet had significantly higher fecal excretion of *Cr* at day 11 post-infection.** Fecal excretion of *Cr* was determined at day 11 post infection for mice fed the basal TWD diet or the TWD diet containing 10% RPS. Data is from four independent experiments combined. Bars are the mean ± SEMn=13-24.

**Suppl. Figure 4. Both infection and diet affected the variance components associated with 16S analysis PCA plots.** JMP Genomics was used to evaluate the factors contributing to the variance of 16S sequences obtained from uninfected and infected mice fed diets containing 0%, 2%, 5%, or 10% RPS. The variance component plot for the cecal samples A) where treatment (dietary RPS) was the predominant component as compared to the feces B) where infection and treatment variance components were nearly equal.

**Suppl. Figure 5. Stack plots of the relative abundance of genera obtained from cecal and fecal samples.** A) genus stack plots for cecal samples obtained 12 days post-infection. B) genus stack plots for fecal samples obtained 6 days post-infection. n= 4-5 mice/group.

**Suppl. Figure 6. PCA Analysis of Gene Expression in Cecum and Distal Colon of *Cr*-Infected Mice.** PCA plots and graphs of variance contributions were generated from RNAseq data for the cecum (A and B) and distal colon (C and D). Plots were generated using JMP Genomics. n=4-5/group.

**Suppl. Figure 7. Venn analysis of shared differentially expressed genes in cecum and distal colon of uninfected mice.** Differentially expressed genes (up-or downregulated) >1.5 fold at a FDR adjusted p value < 0.05) in each tissue in animals fed 10% RPS, were analyzed by Venn analysis using the online tool Venny2.1 (<https://bioinfogp.cnb.csic.es/tools/venny/>). Genes that were upregulated A) or downregulated B) in cecum or upregulated C) or downregulated D) in DC. n=4-5/group.

**Suppl. Figure 8.** **Genes associated with vitamin A (VA) metabolism downregulated by feeding RPS in the DC.** Sixteen genes downregulated by RPS in DC associated with vitamin A (VA) metabolism. The PIN database was used to identify literature-based associations of genes with a defined role in VA metabolism. These genes were remapped into DAVID using KEGG and appear in Suppl. Figure 8, n=4-5/group. Genes in the pathway (mmu00830:Retinol metabolism) with a red star were downregulated by RPS FDRadj p = 0.008.
